# Synthesis and Evaluation of Cytotoxic Activity of RuCp(II) Complexes Bearing (Iso)nicotinic Acid Based Ligands

**DOI:** 10.3390/ph18010097

**Published:** 2025-01-14

**Authors:** Bárbara Marques, Diogo M. Engrácia, João Franco Machado, Jaime A. S. Coelho, Filipa Mendes, Tânia S. Morais

**Affiliations:** 1 Centro de Química Estrutural, Institute of Molecular Sciences, Faculdade de Ciências, Universidade de Lisboa, Campo Grande, 1749-016 Lisboa, Portugal; bsmarques@alunos.fc.ul.pt (B.M.); jmfmachado@ciencias.ulisboa.pt (J.F.M.); jacoelho@ciencias.ulisboa.pt (J.A.S.C.); 2 Centro de Ciências e Tecnologias Nucleares, Instituto Superior Técnico, Universidade de Lisboa, 2695-066 Bobadela LRS, Portugal; diogo.engracia@tecnico.ulisboa.pt (D.M.E.); fmendes@ctn.tecnico.ulisboa.pt (F.M.); 3 Departamento de Química e Bioquímica, Faculdade de Ciências, Universidade de Lisboa, Campo Grande, 1749-016 Lisboa, Portugal; 4 Departmento de Engenharia e Ciências Nucleares, Instituto Superior Técnico, Universidade de Lisboa, 2695-066 Bobadela LRS, Portugal

**Keywords:** ruthenium–cyclopentadienyl, organometallic, (iso)nicotinic acid, DFT, anticancer

## Abstract

Background/Objectives: Cancer remains one of the major challenges of our century. Organometallic ruthenium complexes are gaining recognition as a highly promising group of compounds in the development of cancer treatments. Methods: Building on the auspicious results obtained for [Ru(η^5^-C_5_H_5_)(PPh_3_)(bipy)][CF_3_SO_3_] (TM34), our focus has shifted to examining the effects of incorporating bioactive ligands into the TM34 framework, particularly within the cyclopentadienyl ring. Results: In this study, we report the synthesis and characterization of two new ruthenium(II) complexes with the general formula [Ru(η^5^-C_5_H_4_CCH_3_=R)(PPh_3_)(bipy)][CF_3_SO_3_], where R represents a nicotinic acid derivative (NNHCO(py-3-yl)) (1) or an isoniazid derivative (NNHCO(py-4-yl)) (2). The complexes were fully characterized using a combination of spectroscopic techniques and computational analysis, revealing the presence of *E/Z*-hydrazone isomerism. Stability studies confirmed the robustness of both complexes in biological media, with compound 1 maintaining good stability in buffer solutions mimicking physiological (pH 7.4) and tumor-like (pH 6.8) environments. The cytotoxicity of the complexes was evaluated in vitro in several human cancer cell lines, namely melanoma (A375), alveolar adenocarcinoma (A549), epidermoid carcinoma (A431), and breast cancer (MDA-MB 231). Conclusions: Both compounds exhibited moderate to high cytotoxic activity, with complex 1 showing a greater propensity to induce cell death, particularly in the A431 and MDA-MB 231 cell lines.

## 1. Introduction

Cancer remains one of the most challenging global health problems, responsible for millions of deaths annually, highlighting the urgent need for more effective treatments. Metal-based drugs, such as platinum-based chemotherapeutics, have been a keystone in the treatment of various cancers, but significant side effects and the development of drug resistance often constrain their use [1,2,3,4]. To address these limitations, efforts have increasingly focused on the potential of other metals, including ruthenium, iron, gold, palladium, iridium, and cobalt [5,6,7,8,9,10,11].

Ruthenium complexes have gained significant momentum as prospective anticancer agents, owing to their unique chemical properties, such as redox activity, stability under physiological conditions, lower toxicity, and diverse modes of action, including DNA binding, protein interaction, and the generation of reactive oxygen species [12,13,14,15]. So far, some ruthenium-based complexes have been investigated in phase I and II clinical trials for cancer chemotherapy, yielding promising results. These include NAMI-A (2004, antimetastatic drug); BOLD-100 (2016, advanced gastrointestinal cancers) and its precursor molecules KP1019 and derivative IT-139, formerly known as KP1339 (2006 and 2012, active against primary tumors); and TLD1433 (2018, for anticancer photodynamic therapy) [16,17,18,19,20].

Half-sandwich organometallic ruthenium(II)–arene complexes have also emerged as an auspicious class of compounds for the development of new anticancer agents, offering multiple opportunities to fine-tune their pharmacological properties. By adjusting factors such as their stability, reactivity, cellular uptake, and selective interaction with biological targets, these complexes have the potential to reduce side effects and overcome resistance, making them strong candidates in the quest for more effective cancer treatments [21,22,23,24,25,26,27]. Our group has been focused on the development of organometallic compounds based on the Ru(η^5^-C_5_H_5_) (RuCp) scaffold as prospective anticancer agents. Most of these compounds, which incorporate mono- or bidentate heteroaromatic and phosphane ligands, have demonstrated anticancer and/or antimetastatic properties, both in vitro and in vivo, underscoring their potential as effective anticancer agents [22,28,29,30,31]. The ruthenium complex [RuCp(PPh_3_)(bipy)][CF_3_SO_3_] (TM34), one of the most promising candidates, demonstrates greater cytotoxicity than cisplatin in several cancer cell lines, including breast, ovarian, prostate, and leukemia. This enhanced effectiveness is attributed to a distinct mode of action that targets the cell membrane, Golgi apparatus, and mitochondria, rather than the nuclear DNA, potentially overcoming cisplatin resistance [22,29].

Encouraged by the remarkable results that we achieved with TM34, we have now investigated the impact of introducing bioactive ligands into the TM34 scaffold, specifically within the cyclopentadienyl ring. The bioactive ligands nicotinic acid (vitamin B3) and isoniazid, although traditionally known for their roles in nutrition and tuberculosis treatment, respectively [32,33], have also gained attention in cancer research for their potential anticancer properties [34,35,36,37]. Herein, we report the synthesis and characterization of two new ruthenium(II) complexes with the general formula [Ru(η^5^-C_5_H_4_CCH_3_=R)(PPh_3_)(bipy)][CF_3_SO_3_], where R = NNHCO(py-3-yl) (nicotinic acid derivative) or NNHCO(py-4-yl) (isoniazid derivative). After complete characterization using experimental and computational techniques, their cytotoxicity was determined in vitro in a panel of cancer cell lines: human melanoma (A375), human alveolar adenocarcinoma (A549), human epidermoid carcinoma (A431), and human breast cancer (MDA-MB 231).

## 2. Results and Discussion

### 2.1. Design and Synthesis of New Ru Complexes

**Figure 1:**
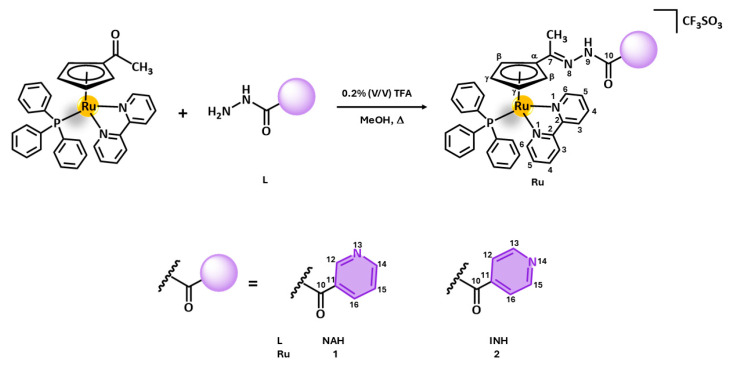
Reaction scheme for the synthesis of [Ru(η^5^-C_5_H_4_CCH_3_=R)(PPh_3_)(bipy)][CF_3_SO_3_], where R = NNHCO(py-3-yl) (**1**) or NNHCO(py-4-yl) (**2**) = pbt. Ligands are numbered for NMR spectral assignment.

Two new complexes with the general formula [Ru(η^5^-C_5_H_4_CCH_3_=R)(PPh_3_)(bipy)][CF_3_SO_3_], where R = NNHCO(py-3-yl) (**1**) or NNHCO(py-4-yl) (**2**), were prepared via the reaction of [Ru(η^5^-C_5_H_4_COCH_3_)(PPh_3_)(bipy)][CF_3_SO_3_] with nicotinic acid hydrazide (NAH) or isoniazid (INH), respectively, in methanol over reflux in the presence of trifluoroacetic acid (0.2% *v*/*v*); see Figure 1. Both complexes were afforded in an *E/Z*-hydrazone isomeric mixture (4:1) with high purity upon recrystallization via the slow diffusion of diethyl ether in dichloromethane solutions, with moderate yields (62% and 73%, respectively). Moreover, 1D/2D NMR, FTIR, and UV–vis spectroscopies and elemental analysis confirmed the proposed structures.

NMR characterization was carried out in DMSO-*d_6_* by ^1^H, APT-^13^C{^1^H}, and ^31^P{^1^H} experiments, complemented by 2D experiments (COSY, NOESY, HSQC, and HMBC). The numbering of the C and H atoms for assignment purposes is depicted in Figure 1. The spectra of complexes **1** and **2** did not show the NH_2_ resonance of the hydrazide group comparatively to free NAH or INH, respectively, suggesting successful conjugation through the hydrazone bond. As a result of C=N bond formation (electro-attractive), the deshielding of the H9 proton was observed for both complexes (Δδ = 0.7 ppm). Additionally, upon conjugation, the shielding of the -CH_3_ protons in complexes **1** and **2** occurred, comparatively to the precursor complex [Ru(η^5^-C_5_H_4_COCH_3_)(PPh_3_)(bipy)][CF_3_SO_3_] (Δ_δ_ = 0.1 ppm), due to the substitution of the neighbor oxygen atom for a less electronegative nitrogen atom. The deshielding of H-γ protons (Δ_δ_ = 0.2 ppm) with the simultaneous shielding of H-β protons (Δ_δ_ ≈ 0.3 ppm) of the Cp ring in both complexes **1** and **2** was also observed upon conjugation, as a result of changes in electronic flow from the metal center to Cp induced by {NNHCO(py-3-yl)} or {NNHCO(py-4-yl)} fragments, in accordance with the UV–vis studies discussed below. No further significant modifications in the chemical shifts of the remaining resonances were observed (in PPh_3_ and bipy coligands, for instance), as expected. A second set of resonances, at a ratio of 4/1, was identified in the NMR spectra of both complexes, suggesting the existence of *E/Z*-hydrazone isomerism. Both isomers presented quite similar chemical shifts (with part of them being overlapped), with the exception of the H-γ and H-β protons from Cp and the H9 proton from hydrazone, which presented a difference of ≈0.1, ≈0.3, and ≈0.2 ppm, respectively. These protons are more affected given the proximity to the imine bond responsible for geometric isomerism. NOESY experiments were conducted, aimed at identifying the major isomer, but without success. Thus, further DFT calculations were performed to gain insights into the structure and thermodynamic stability of the *E*/*Z*-isomers and support our findings (see Section 2.2).

The ^13^C NMR spectra of compounds **1** and **2** follow a similar pattern to the ^1^H spectra. The shielding of C7 in both complexes comparatively to [Ru(η^5^-C_5_H_4_COCH_3_)(PPh_3_)(bipy)][CF_3_SO_3_] (Δ_δ_ ≈ 40 ppm) once more indicates successful conjugation through hydrazone bond formation. ^31^P NMR shows two peaks for the phosphane ligand, one for each isomer, in accordance with the complexes’ symmetry, once more evidencing *E/Z*-isomerism. There were no significant differences between the two compounds and the precursor complex, as there were no changes in the coordination sphere.

For both complexes, the FTIR spectra present the characteristic bands of the cyclopentadienyl and phenyl rings (υ_C-H_ at 3030–3130 cm^−1^; υ_C=C_ at 1430–1550 cm^−1^) and the sulfonate group of the counter anion [CF_3_SO_3_]^−^ (υ_S=O_ at 1250–1280 cm^−1^). The conjugation of the NAH/INH ligands is confirmed by the appearance of new bands in both complexes’ spectra, such as the ones corresponding to the N-H bond (υ_N-H_ at 3200–2300 cm^−1^; δ_N-H_ at 1603–1605 cm^−1^).

**Figure 2:**
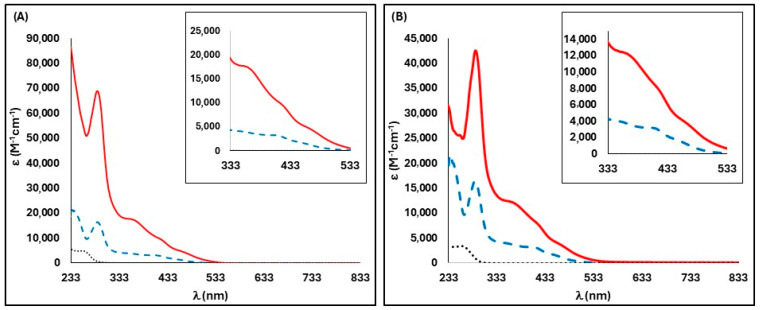
Electronic spectra of (**A**) [Ru(η^5^-C_5_H_4_CCH_3_=R1)(PPh_3_)(bipy)][CF_3_SO_3_], R1 = NNHCO(py-3-yl) (**1**) (**−**) in dichloromethane, compared with the precursor [Ru(η^5^-C_5_H_4_COCH_3_)(PPh_3_) (bipy)][CF_3_SO_3_] (---) and free ligand NAH (···); (**B**) [Ru(η^5^-C_5_H_4_CCH_3_=R2)(PPh_3_)(bipy)][CF_3_SO_3_], R2 = NNHCO(py-4-yl) (**2**) (**−**) in dichloromethane, compared with the precursor [Ru(η^5^-C_5_H_4_COCH_3_)(PPh_3_) (bipy)][CF_3_SO_3_] (---) and free ligand INH (···). Inset: detail of charge transfer bands.

The electronic spectra of complexes **1** and **2** were acquired in 1.50 × 10^−5^ to 2.90 × 10^−5^ mol dm^−3^ dichloromethane solutions, at room temperature. Both complexes showed similar behavior. Figure 2 presents the electronic spectra of these two compounds. Both new complexes show a band with a high molar absorption coefficient at 280 nm or 288 nm, respectively, corresponding to intra-ligand π–π* transitions. A shoulder is present at 373 nm (**1**) or 366 nm (**2**), attributed to metal–ligand charge transference (MLCT) between the Ru center and the pyridine moiety of the derivatized cyclopentadienyl ring. Two other shoulders are present, at 424 nm and 467 nm for compound **1** or 427 nm and 478 nm for compound **2**, which are derived from the MLCT between the metallic center and the bipy and PPh_3_ ligands.

### 2.2. Computational Study on the Structures of the Complexes

**Figure 3:**
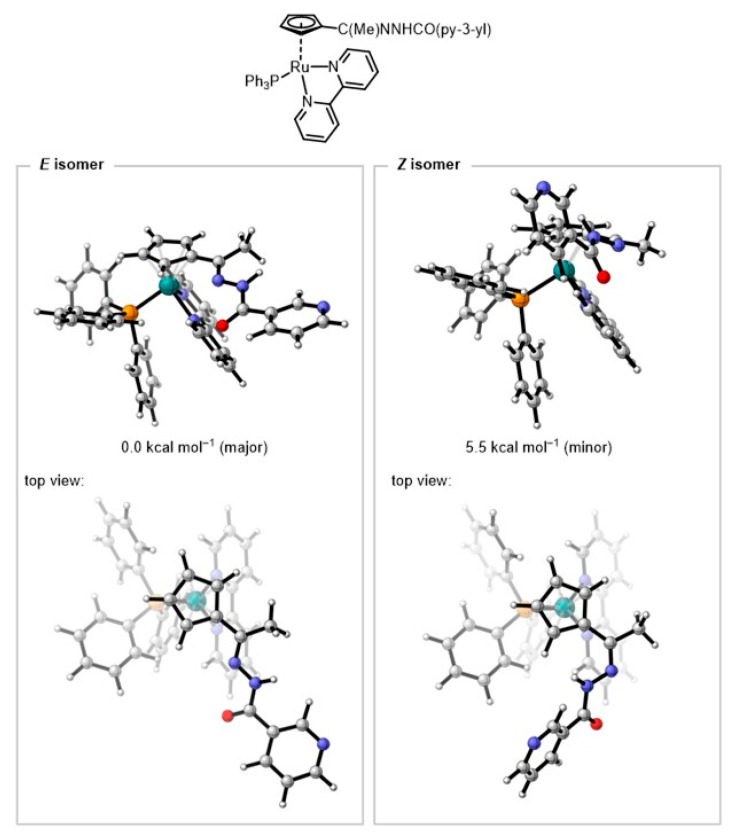
Low-lying geometries and relative Gibbs free energies (kcal mol^−1^) of E- and Z-isomers of [Ru(η^5^-C_5_H_4_CCH_3_NNHCO(py-3-yl))(PPh_3_)(bipy)].

Density functional theory (DFT) calculations were performed to obtain insights into the structures of the *E/Z*-isomers of [Ru(η^5^-C_5_H_4_CCH_3_NNHCO(py-3-yl))(PPh_3_)(bipy)] and to determine the origin of the thermodynamic stability. The DFT-computed structures of the new complexes were based on the previously reported experimental X-ray structure of the precursor complex [Ru(η^5^-C_5_H_4_COCH_3_)(PPh_3_)(bipy)] [31]. Calculations at the B3PW91/6-311++G(2d,2p); SDD(Ru)/PCM(DMSO)//B3PW91/6-31G(d,p); SDD(Ru) level of theory indicate that the *Z*-isomer is 5.5 kcal mol^−1^ (Gibbs free energy) higher in energy, suggesting that the *E*-isomer is the major product obtained. Energy decomposition analysis reveals that the difference in stability is mainly determined by the interaction energy between the Ru(PPh_3_)(bipy) core and the substituted cyclopentadienyl ligands, as the distortions in both components are minimal (Figure 3 and Figures S1–S3).

### 2.3. Aqueous and pH Stability of the Complexes

The stability of both complexes was evaluated in an aqueous solution prior to their biological evaluation to ensure the structures’ robustness and suitability for a biomedical application. DMSO is a widely established solvent, used as a co-solvent in biological assays due to its ability to effectively dissolve a wide range of compounds; however, it is also a strongly coordinated solvent. Therefore, we examined the stability of both complexes in DMSO and cell culture media (DMSO/DMEM) over 24 h by UV–vis spectrophotometry at room temperature. These results are summarized in Figure S4 and clearly show that no significant alterations in the electronic spectra of both compounds were observed during the 24 h period, in terms of the number of bands, intensity, and λmax (variation < 10% after 24 h) under the various conditions tested, confirming the stability of the complexes. It is important to note that minimal precipitation was observed over time at the concentrations of complex **1** required for this evaluation, resulting in a decrease in the π-π* and MLCT bands; however, this was not seen at the concentrations used in the subsequent NMR studies or in the biological evaluation, which were in the micromolar range for the latter.

**Figure 4:**
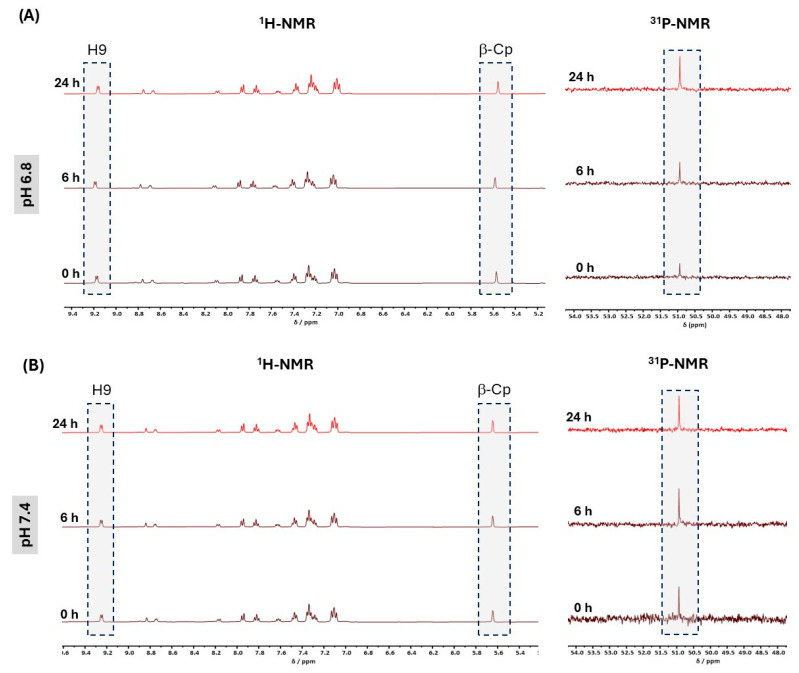
Evaluation of stability of compound **1** in ACN/D_2_O phosphate buffer solution, followed by ^1^H- and ^31^P{^1^H}-NMR spectroscopy over 24 h at pH 6.8 (**A**) and pH 7.4 (**B**).

Both complexes contain hydrazones, which are known to hydrolyze in a pH-dependent manner, possibly affecting their stability and biological activity in different environments. Thus, to understand this behavior and predict their performance in physiological conditions, the stability of complex **1** was also evaluated at pH values of 7.4 (mimicking healthy tissues and bloodstream) and 6.8 (mimicking tumoral microenvironment), by NMR spectroscopy in a D_2_O phosphate buffer solution (ACN/Buffer) over 24 h. Compound **2** was not evaluated because its structure is identical to that of compound **1**, and the surrounding environment of the hydrazone function is the same, suggesting that it is likely to exhibit similar behavior. Figure 4 gathers the ^1^H and ^31^P{^1^H} NMR spectra of the compound and clearly shows that **1** is stable at both pH values over time, as there are no changes in the number of peaks or in their chemical shift values.

### 2.4. Evaluation of the Cytotoxicity of the Complexes

The antiproliferative properties of the two ligands (NAH and INH), the precursor complex, and two novel Ru complexes (**1** and **2**) were assayed by monitoring their ability to inhibit cell growth using a panel of human cancer cell lines of different origins. Cytotoxic activity was determined on human melanoma (A375), human alveolar adenocarcinoma (A549), human epidermoid carcinoma (A431), and human breast cancer (MDA-MB 231) cell lines by the CellTiter-Glo^®^ Viability Assay, which indicates cell viability through ATP measurement. For comparison, the activity of different concentrations of DMSO was also tested in parallel in the same cellular models. Using an appropriate range of concentrations (50–0.01 µM), dose–response curves after 48 h incubation were obtained. The calculated IC_50_ for the compounds from the experimental values is presented in Table 1.

The precursor complex generally presents lower IC_50_ values than the novel complexes, except for the A375 cell line, which has slightly higher sensitivity to **1** and **2** than the precursor. In the case of the A431 cell line, **1** and the precursor present IC_50_ values lower than **2**. Between the two novel complexes, **1** generally induces a higher level of cell death, especially on the A43I and MDA-MB 231 cell lines. The IC_50_ values of the ligands are high across all cell lines, demonstrating no relevant cytotoxicity.

The precursor and the novel complexes have minimal differences and therefore the addition of the (iso)nicotinic acid-based ligands on the Cp ring does not seem to significantly impact the cytotoxicity of the metal complexes compared with the original metal complex. The IC50 values found for the new complexes are within the same range as those of other RuCp and Ru–arene complexes [22,29,31,38].

## 3. Materials and Methods

### 3.1. General Procedures

All syntheses were carried out under a dinitrogen atmosphere using Schlenk techniques. The chemicals and solvents were of analytical grade and used without further purification, except for dichloromethane and n-hexane, which were dried/purified before being used with an MBraun SPS-800 solvent purification system. Starting materials [Ru(η^5^-C_5_H_4_COCH_3_)(PPh_3_)_2_Cl] and [Ru(η^5^-C_5_H_4_COCH_3_)(bipy)][CF_3_SO_3_] were synthesized as previously reported [31].

^1^H, APT-^13^C{^1^H}, and ^31^P{^1^H} NMR spectra were recorded in (CD_3_)_2_SO on a Bruker Avance 400 spectrometer (400.13 MHz, 100.62 MHz, or 161.97 MHz, respectively) at the probe temperature. Chemical shifts (δ) are reported in parts per million (ppm), downfield from internal Me_4_Si at 0.00 ppm for ^1^H and ^13^C NMR or from external standard 85% H_3_PO_4_ for ^31^P{^1^H} NMR. Resonances were unambiguously assigned with the assistance of 2D NMR experiments (COSY, NOESY, HSQC, and HMBC). Abbreviations: s = singlet; d = doublet; t = triplet; m = multiplet; *J* = coupling constant.

FTIR spectra were recorded on a THERMO NICOLET 6700 FTIR spectrophotometer in KBr pellets (4000–400 cm^−1^). Only the most significant bands are cited in the text. Electronic spectra were recorded in dichloromethane at room temperature on a Jasco V-660 spectrophotometer in the 233–900 nm range, using quartz cuvettes with a 1 cm optical path. High-resolution mass spectra were recorded in a Dionex Ultimate 3000 UHPLC+ system equipped with a multiple-wavelength detector, using an imChem Surf C18 TriF 100A 3 μm 100 × 2.1 mm column connected to a Thermo Scientific Q Exactive hybrid quadrupole–Orbitrap mass spectrometer (Thermo ScientificTM Q ExactiveTM Plus, Waltham, MA, USA) and were processed using the MestReNova 14.2 software package. Elemental analyses were performed at the Laboratório de Análises at Instituto Superior Técnico, using a Fisons Instruments EA1108 system. Data acquisition, integration, and handling were performed with the software package EAGER-200 (Carlo Erba Instruments, Cornaredo (Milano), Italy).

### 3.2. Synthesis of Ruthenium Complexes

**[Ru(η^5^-C_5_H_4_CCH_3_=R1)(PPh_3_)(bipy)][CF_3_SO_3_], R1 = NNHCO(py-3-yl) (1)**

NH_2_NHCO(py-3-yl) (0.05 g, 0.375 mmol) was added to a stirring solution of [Ru(η^5^-C_5_H_4_COCH_3_)(bipy)][CF_3_SO_3_] (0.19 g, 0.25 mmol) in methanol (30 mL), in the presence of trifluoroacetic acid (60 μL). The orange mixture was refluxed for 5 h and monitored by ^1^H and ^31^P NMR. After cooling to room temperature, the solvent was evaporated under a vacuum. The product was washed with n-hexane and recrystallized from dichloromethane/diethyl ether, forming a crystalline orange powder. Yield: 62%. ^1^H NMR [(CD_3_)_2_SO, Me_4_Si, δ/ppm]: 10.69 (s, 1H, H9); 9.33 (m, 2H, H6); 8.93 (m, 1H, H12); 8.75 (m, 1H, H14); 8.19 (m, 2H, H3); 8.16 (m, 1H, H16); 7.87 (m, 2H, H4); 7.56 (m, 1H, H15); 7.40 (m, 3H, *p*-PPh3); 7.34 (m, 2H, H5); 7.29 (m, 6H, *m*-PPh3); 6.93 (m, 6H, *o*-PPh3); 5.50 (s, 2H, β-Cp); 4.74 (s, 2H, γ-Cp); 1.47 (s, 3H, -CH_3_). ^13^C NMR [(CD_3_)_2_SO, Me_4_Si δ/ppm]: 162.00 (-C=O, C10); 155.86 (C6); 155.11 (C2); 153.49 (C7); 151.78 (C14); 148.44 (C12); 136.55 (C4); 135.93 (C16); 132.55 (*o*-PPh_3_); 131.02 (*i*-PPh_3_); 130.18 (*p*-PPh_3_); 129.85 (C11); 128.55 (*m*-PPh_3_); 125.37 (C5); 123.60 (C15); 123.21 (C4); 91.68 (α-Cp); 80.00 (β-Cp); 76.34 (γ-Cp); 14.82 (CH_3_). ^31^P{^1^H}NMR [(CD_3_)_2_SO, H3PO4, δ/ppm]: 50.50 (s, PPh_3_). FTIR [KBr, cm^−1^]: 3275–3300 (υ_N-H_, hydrazone); 3030–3130 (υ_C-H_, phenyl and Cp rings); 1660 (υ_C=O_, hydrazone); 1605 (δ_N-H_, hydrazone); 1430–1550 (υ_C=C_, phenyl rings and υ_C=N_, hydrazone); 1250–1280 (υ_S=O_, [CF_3_SO_3_]^−^). HRMS (ESI-MS): *m*/*z* calcd for C_41_H_35_RuN_5_OP [M]+ = 746.1628, found = 746.1625. Elemental analysis (%) found: C, 55.35; H, 3.70; N, 7.95; S, 3.00. Calc. for C_42_H_35_N_5_SPF_3_O_4_Ru·0.3CH_2_Cl_2_ (920.35): C, 55.20; H, 3.90; N, 7.61; S, 3.48%. U*V*/*V*is (CH_2_Cl_2_) λ_max_/nm (ε/M^−1^cm^−1^): 478 (*sh*); 427 (*sh*); 366 (*sh*); 288 (68894).

**[Ru(η^5^-C_5_H_4_CCH_3_=R2)(PPh_3_)(bipy)][CF_3_SO_3_], R2 = NNHCO(py-4-yl) (2)**

NH_2_NHCO(py-4-yl) (0.05 g, 0.375 mmol) was added to a stirring solution of [Ru(η^5^-C_5_H_4_COCH_3_)(bipy)][CF_3_SO_3_] (0.19 g, 0.25 mmol) in methanol (30 mL), in the presence of trifluoroacetic acid (60 μL). The mixture was heated to reflux for 4 h, changing the color from brownish orange to bright orange. After refluxing, the solvent was evaporated under a vacuum and the product was recrystallized from dichloromethane/diethyl ether, resulting in a crystalline orange powder. Yield: 73%.^1^H NMR [(CD_3_)_2_SO, Me_4_Si, δ/ppm]: 10.79 (s, 1H, H9); 9.33 (d, 2H, *J* = 5.2 Hz, H6); 8.78 (m, 2H, H13 + H15); 8.19 (d, 2H, *J* = 8.0 Hz, H3); 7.87 (m, 2H, H4); 7.78 (d, 2H, *J* = 4.9 Hz, H12 + H16); 7.40 (m, 3H, *p*-PPh3); 7.34 (m, 2H, H5); 7.29 (m, 6H, *m*-PPh3); 6.93 (m, 6H, *o*-PPh3); 5.52 (s, 2H, β-Cp); 4.74 (s, 2H, γ-Cp); 1.47 (s, 3H, -CH_3_). ^13^C NMR [(CD_3_)_2_SO, Me_4_Si, δ/ppm]: 161.71 (C10); 155.85 (C6); 155.12 (C2); 154.84 (C7); 149.22 (C13); 142.05 (C11); 136.57 (C4); 132.57 (*o*-PPh_3_); 131.00 (*i*-PPh_3_); 130.18 (*p*-PPh_3_); 128.55 (*m*-PPh_3_); 125.39 (C5); 123.23 (C3); 122.26 (C12); 90.97 (α-Cp); 80.35 (β-Cp); 76.38 (γ-Cp); 14.94 (CH_3_). ^31^P{^1^H} NMR [(CD_3_)_2_SO, H3PO4, δ/ppm]: 50.37 (s, PPh_3_). FTIR [KBr, cm^−1^]: 3200–3280 (υ_N-H_, hydrazone); 3050–3120 (υ_C-H_, phenyl and Cp rings); 1676 (υ_C=O_, hydrazone); 1603 (δ_N-H_, hydrazone); 1430–1550 (υ_C=C_, phenyl rings and υ_C=N_, hydrazone); 1250–1280 (υ_S=O_, [CF_3_SO_3_]^−^). HRMS (ESI-MS): *m*/*z* calcd for C_41_H_35_RuN_5_OP [M]+ = 746.1628, found = 746.1625. Elemental analysis (%) found: C, 52.20; H, 3.40; N, 7.05; S, 3.00. Calc. for C_42_H_35_N_5_SPF_3_O_4_Ru·1.1CH_2_Cl_2_ (988.29): C, 52.38; H, 3.79; N, 7.09; S, 3.24. U*V*/*V*is (CH_2_Cl_2_) λ_max_/nm (ε/M^−1^cm^−1^): 467 (*sh*); 424 (*sh*); 373 (*sh*); 289 (42552).

### 3.3. Stability in Aqueous Medium and pH Effect

The stability of complexes **1** and **2** in organic (100% DMSO) and aqueous (5% DMSO/95% DMEM+GlutaMAX-I™) solutions (2.46 × 10^−5^ M–7.93 × 10^−5^ M) was evaluated at room temperature by UV–vis spectroscopy over 24 h, on a Jasco V-560 spectrometer using quartz cuvettes with a 1 cm optical path (268–900 nm). The spectra were acquired at t = 0 h, 0.15 h, 0.30 h, 0.45 h, 1 h, 2 h, 3 h, 4 h, 5 h, and 24 h. The variation in the maximum absorbance over time was calculated for the π-π* and charge transfer bands in the range of 290–370 nm.

Additionally, the stability of complex **1** was also evaluated in an aqueous solution (14% ACN/86% phosphate buffer 10 mM in D_2_O) at pH values that mimicked the tumor microenvironment (6.8) or healthy tissue/bloodstream (7.4) by ^1^H and ^31^P NMR. Solutions of **1** (2.23 × 10^−3^ M) were monitored for 24 h (t = 0 h, 1 h, 2 h, 3 h, 4 h, 5 h, 6 h, 24 h).

Samples were kept at room temperature and protected from light between measurements.

### 3.4. Computational Details

Density functional theory (DFT) calculations were performed using the Gaussian 16 software package [39], and structural representations were generated with CYLview20 [40]. Geometry optimizations were carried out using the hybrid GGA functional B3PW91 and the SDD basis set for the Ru atom (pseudopotential) and the valence double-zeta 6-31G(d,p) basis set for all other atoms. All of the optimized geometries were verified by frequency computations as minima (zero imaginary frequencies). Single-point energy calculations on the optimized geometries were then evaluated using the same functional and the SDD basis set for the Ru atom and the 6-311++G(2d,2p) basis set for all other atoms, with solvent effects (dimethylsulfoxide) calculated by means of the polarizable continuum model (PCM). Free energy values were derived from the electronic energy corrected by using the thermal and entropic corrections based on structural and vibration frequency data.

### 3.5. Cytotoxicity Assay

The cancer cell lines A375, A431, A549, and MDA-MB231 were cultured in Dulbecco’s Modified Eagle Medium (DMEM) with GlutaMAX™ supplemented with 10% fetal bovine serum (FBS). Cell lines were grown at 37 °C in a humidified atmosphere containing 5% CO_2_. All cell lines were tested for mycoplasma using the LookOut^®^ mycoplasma PCR Detection Kit. Stock solutions of the compounds tested (5 mM) were prepared in 100% DMSO. Dilutions were prepared in complete DMEM in order to obtain the desired concentrations. As a control, wells were incubated with a complete medium and with DMSO. The CellTiter-Glo^®^ 3D Cell Viability Assay kit was used to assess cell viability after incubation with the compounds for 48 h. One hundred µL of culture medium was removed from each well and replaced with 40 µL of the CellTiter-Glo^®^ 3D Cell Viability Assay. After 15 min incubation with agitation, 100 µL was transferred to a 96-well white bottom plate, and the luminescence was measured using a microplate reader (Varioskan™ LUX multimode microplate reader, Thermo Scientific). All compounds were tested in at least two independent experiments, each comprising three/four replicates per concentration. The IC_50_ and SD values were calculated from dose–response curves analyzed using the GraphPad Prism software (version 10.0).

## 4. Conclusions

For the first time, two Ru(II)Cp complexes incorporating bioactive ligands, with the general formula [Ru(η^5^-C_5_H_4_CCH_3_=R)(PPh_3_)(bipy)][CF_3_SO_3_], where R = NNHCO(py-3-yl) (nicotinic acid derivative) or NNHCO(py-4-yl) (isoniazid derivative), were synthesized and evaluated for their cytotoxic activity. Both compounds were fully characterized using spectroscopic methods, and NMR analysis revealed two sets of resonances in a 4:1 ratio, indicative of *E/Z*-hydrazone isomerism. This observation was consistent with the results obtained from density functional theory (DFT) calculations.

The stability of both compounds was confirmed in a biological medium (DMSO/DMEM), with compound **1** also proving stable in buffer solutions at pH 7.4 and 6.8, simulating the conditions of healthy tissue/bloodstream and the tumor microenvironment, respectively.

In terms of cytotoxicity, both compounds demonstrated moderate to high activity in the A375, A549, A431, and MDA-MB 231 cancer cell lines. Notably, complex **1** exhibited a stronger ability to induce cell death compared to complex **2**, particularly in A431 and MDA-MB 231 cells.

Interestingly, the precursor complex [Ru(η^5^-C_5_H_4_COCH_3_)(bipy)][CF_3_SO_3_] generally exhibited lower IC_50_ values than the new complexes, with the A375 cell line displaying slightly higher sensitivity to the novel compounds.

Overall, the minimal differences in the IC_50_ values between the new complexes and the precursor suggest that incorporating (iso)nicotinic acid-based ligands into the Cp ring does not significantly enhance the cytotoxicity of the metal complexes. Nonetheless, this study provides a valuable framework for future investigations, offering a platform for the development of novel metal-based therapeutics with the potential for improved biological activity and therapeutic efficacy.

## Figures and Tables

**Table 1:** IC_50_ values of the precursor, **1**, **2**, and the ligands NAH and INH on a panel of cancer cell lines after 48 h incubation.

	IC_50_ Values (μM) + SD			
Compound	A375	A431	A549	MDA-MB 231
Precursor	5.18 ± 0.63	0.44 ± 0.19	1.62 ± 0.45	0.20 ± 0.16
**1**	4.45 ± 1.40	0.31 ± 0.15	1.92 ± 0.91	0.36 ± 0.34
**2**	4.56 ± 1.45	2.90 ± 0.96	2.37 ± 1.39	1.86 ± 2.64
NAH	>50	>50	>50	>50
INH	>50	>50	>50	>50

## Data Availability

Data is contained within the article and Supplementary Materials.

## Supplementary Materials

The following supporting information can be downloaded at: https://www.mdpi.com/article/10.3390/ph18010097/s1, Figure S1: Workflow for the optimization of new complexes based on the structure of [Ru(η^5^-C_5_H_4_COCH_3_)(PPh_3_)(bipy)]; Figure S2: Low-lying geometries and relative Gibbs free energies (kcal mol^−1^) of E- and Z-isomers of C_5_H_4_CCH_3_NNHCO(py-3-yl) anion; Figure S3: Energy decomposition analysis for *E/Z* isomers of [Ru(η^5^-C_5_H_4_CCH_3_NNHCO(py-3-yl))(PPh_3_)(bipy)]. *E/Z* energy difference (∆E_total_ *E/Z*); distortion energy for *E/Z* from cyclopentadienyl anion ligand (∆E_dist_ CpC(Me)NNHCO(py-3-yl)); distortion energy for *E/Z* from [Ru(PPh_3_)(bpy)]^2+^ (∆E_dist_ [Ru(PPh_3_)(bpy)]). Calculated energies were computed at B3PW91/6-311++G(2d,2p); SDD(Ru)/PCM (DMSO) level and are given in kcal mol^−1^; Figure S4: Stability studies followed by UV–vis spectroscopy over 24 h: (A) compound 1 in DMSO; (B) compound 1 in cellular media (DMSO/DMEM); (C) compound 2 in DMSO; (D) compound 2 in cellular media (DMSO/DMEM). (E) Maximum absorbance variation (%) over time for compound 1 in DMSO (▲, λ = 361 nm); compound 1 in cellular media (●, λ = 367 nm); compound 2 in DMSO (♦, λ = 367 nm); compound 2 in cellular media (▲, λ = 368 nm) (see experimental section for details); Figure S5: ^1^H NMR spectrum of [Ru(η^5^-C_5_H_4_CCH_3_=R1)(PPh_3_)(bipy)][CF_3_SO_3_], R1 = NNHCO(py-3-yl) (1) in DMSO-d_6_; Figure S6: ^31^P NMR spectrum of [Ru(η^5^-C_5_H_4_CCH_3_=R1)(PPh_3_)(bipy)][CF_3_SO_3_], R1 = NNHCO(py-3-yl) (1) in DMSO-d_6_; Figure S7: COSY NMR spectrum of [Ru(η^5^-C_5_H_4_CCH_3_=R1)(PPh_3_)(bipy)][CF_3_SO_3_], R1 = NNHCO(py-3-yl) (1) in DMSO-d_6_; Figure S8: ^13^C NMR spectrum of [Ru(η^5^-C_5_H_4_CCH_3_=R1)(PPh_3_)(bipy)][CF_3_SO_3_], R1 = NNHCO(py-3-yl) (1) in DMSO-d_6_; Figure S9: HSQC NMR spectrum of [Ru(η^5^-C_5_H_4_CCH_3_=R1)(PPh_3_)(bipy)][CF_3_SO_3_], R1 = NNHCO(py-3-yl) (1) in DMSO-d_6_; Figure S10: HMBC NMR spectrum of [Ru(η^5^-C_5_H_4_CCH_3_=R1)(PPh_3_)(bipy)][CF_3_SO_3_], R1 = NNHCO(py-3-yl) (1) in DMSO-d_6_; Figure S11: ^1^H NMR spectrum of [Ru(η^5^-C_5_H_4_CCH_3_=R2)(PPh_3_)(bipy)][CF_3_SO_3_], R2 = NNHCO(py-4-yl) (2) in DMSO-d_6_; Figure S12: ^31^P NMR spectrum of [Ru(η^5^-C_5_H_4_CCH_3_=R2)(PPh_3_)(bipy)][CF_3_SO_3_], R2 = NNHCO(py-4-yl) (2) in DMSO-d6; Figure S13: COSY NMR spectrum of [Ru(η^5^-C_5_H_4_CCH_3_=R2)(PPh_3_)(bipy)][CF_3_SO_3_], R2 = NNHCO(py-4-yl) (2) in DMSO-d_6_; Figure S14: ^13^C NMR spectrum of [Ru(η^5^-C_5_H_4_CCH_3_=R2)(PPh_3_)(bipy)][CF_3_SO_3_], R2 = NNHCO(py-4-yl) (2) in DMSO-d_6_; Figure S15. HSQC NMR spectrum of [Ru(η^5^-C_5_H_4_CCH_3_=R2)(PPh_3_)(bipy)][CF_3_SO_3_], R2 = NNHCO(py-4-yl) (2) in DMSO-d_6_; Figure S16. HMBC NMR spectrum of [Ru(η^5^-C_5_H_4_CCH_3_=R2)(PPh_3_)(bipy)][CF_3_SO_3_], R2 = NNHCO(py-4-yl) (2) in DMSO-d_6_; Figure S17. ^1^H NMR spectrum of [Ru(η5-C_5_H_4_CH_3_)(PPh_3_)(bipy)][CF_3_SO_3_] in DMSO-d_6_; Figure S18. ^31^P NMR spectrum of [Ru(η^5^-C_5_H_4_CH_3_)(PPh_3_)(bipy)][CF_3_SO_3_] in DMSO-d_6_; Figure S19. ^1^H NMR spectrum of NAH in DMSO-d_6_; Figure S20. ^1^H NMR spectrum of INH in DMSO-d_6_; Figure S21: FTIR spectrum of [Ru(η^5^-C_5_H_4_CCH_3_=R1)(PPh_3_)(bipy)][CF_3_SO_3_], R1 = NNHCO(py-3-yl) (1) in KBr; Figure S22: FTIR spectrum of [Ru(η^5^-C_5_H_4_CCH_3_=R2)(PPh_3_)(bipy)][CF_3_SO_3_], R2 = NNHCO(py-4-yl) (2) in KBr. Figure S23. (A) High-resolution ESI-MS spectrum of complex 1; calculated (B) and found (C) isotopic pattern. Figure S24. (A) High-resolution ESI-MS spectrum of complex 2; calculated (B) and found (C) isotopic pattern.
