# Supplementary material for: Synthesis and Evaluation of Cytotoxic Activity of RuCp(II) Complexes Bearing (Iso)nicotinic Acid Based Ligands
Source: Pharmaceuticals (Basel). 2025 Jan 14;18(1):97. doi: 10.3390/ph18010097 (PMC11768749; doi:10.3390/ph18010097)
Supplement: Supplementary file 1 [file pharmaceuticals-18-00097-s001.zip › pharmaceuticals-3356524-supplementary.pdf]

Synthesis and Evaluation of Cytotoxic Activity of RuCp(II) Complexes Bearing (Iso)nicotinic Acid Based Ligands

Bárbara Marques[1], Diogo M. Engrácia[2], João Franco Machado[1], Jaime A. S. Coelho[1,3], Filipa Mendes[3,4] and Tânia S. Morais[1,3*]

SUPPORTING INFORMATION

COMPUTATIONAL STUDIES

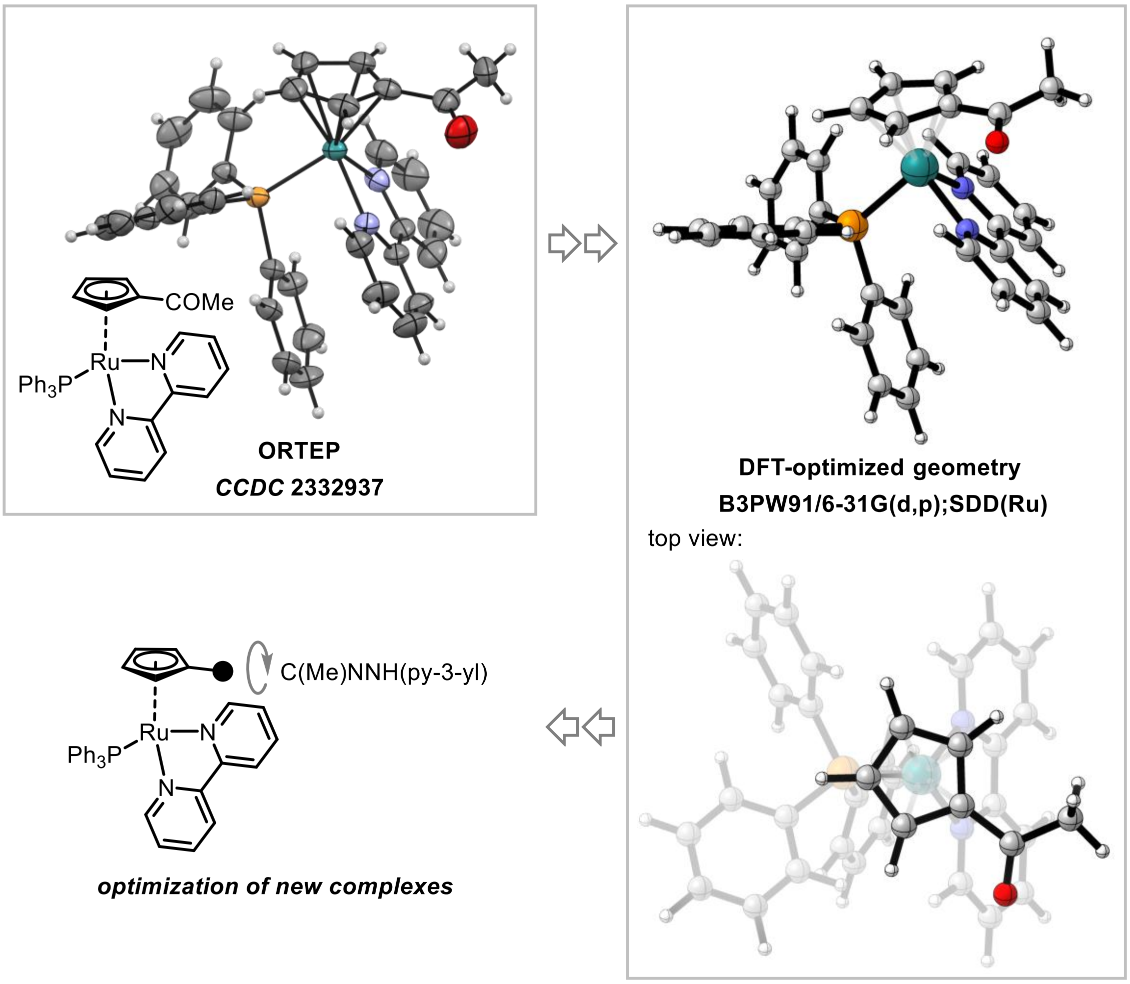

Figure S1. Workflow for the optimization of new complexes based on the structure of $[Ru(\eta^5\text{-}C_5H_4COCH_3)(PPh_3)(bipy)]$.

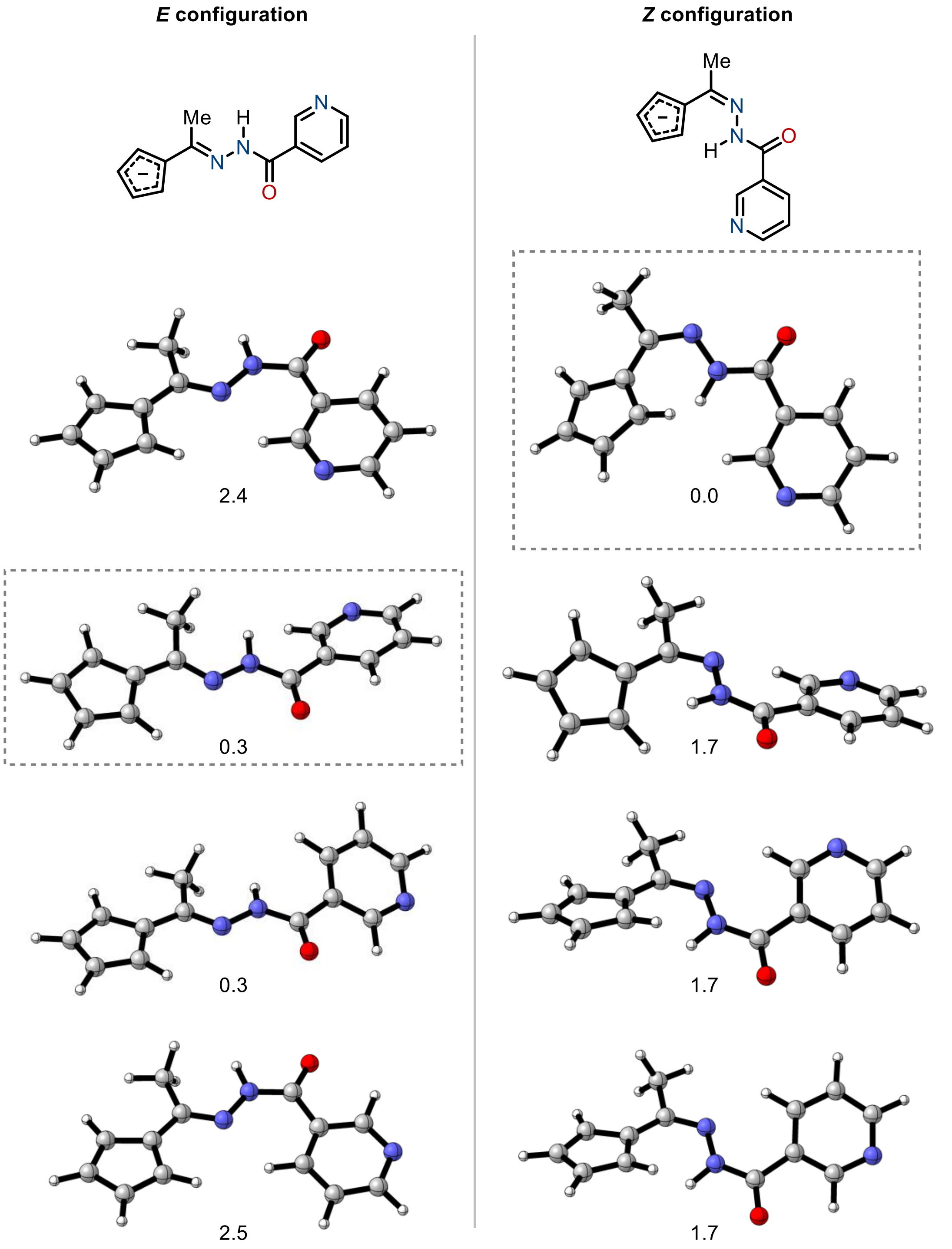

Figure S2. Low-lying geometries and relative Gibbs free energies (kcal mol^{-1}) of E- and Z-isomers of $C_5H_4CCH_3NNHCO$(py-3-yl) anion.

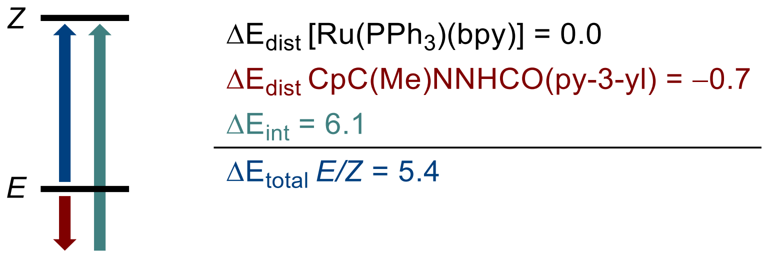

Figure S3. Energy decomposition analysis for E/Z isomers of [Ru(η^5-$C_5H_4CCH_3$NNHCO(py-3-yl))(PPh_3)(bipy)]. E/Z energy difference (ΔE_{total} E/Z); distortion energy for E/Z from cyclopentadienyl anion ligand (ΔE_{dist} CpC(Me)NNHCO(py-3-yl)); distortion energy for E/Z from [Ru(PPh_3)(bpy)]$^{2+}$ (ΔE_{dist} [Ru(PPh_3)(bpy)]). Calculated energies were computed at B3PW91/6-311++G(2d,2p); SDD(Ru)/PCM (DMSO) level and are given in kcal mol^{-1}.

STABILITY STUDIES

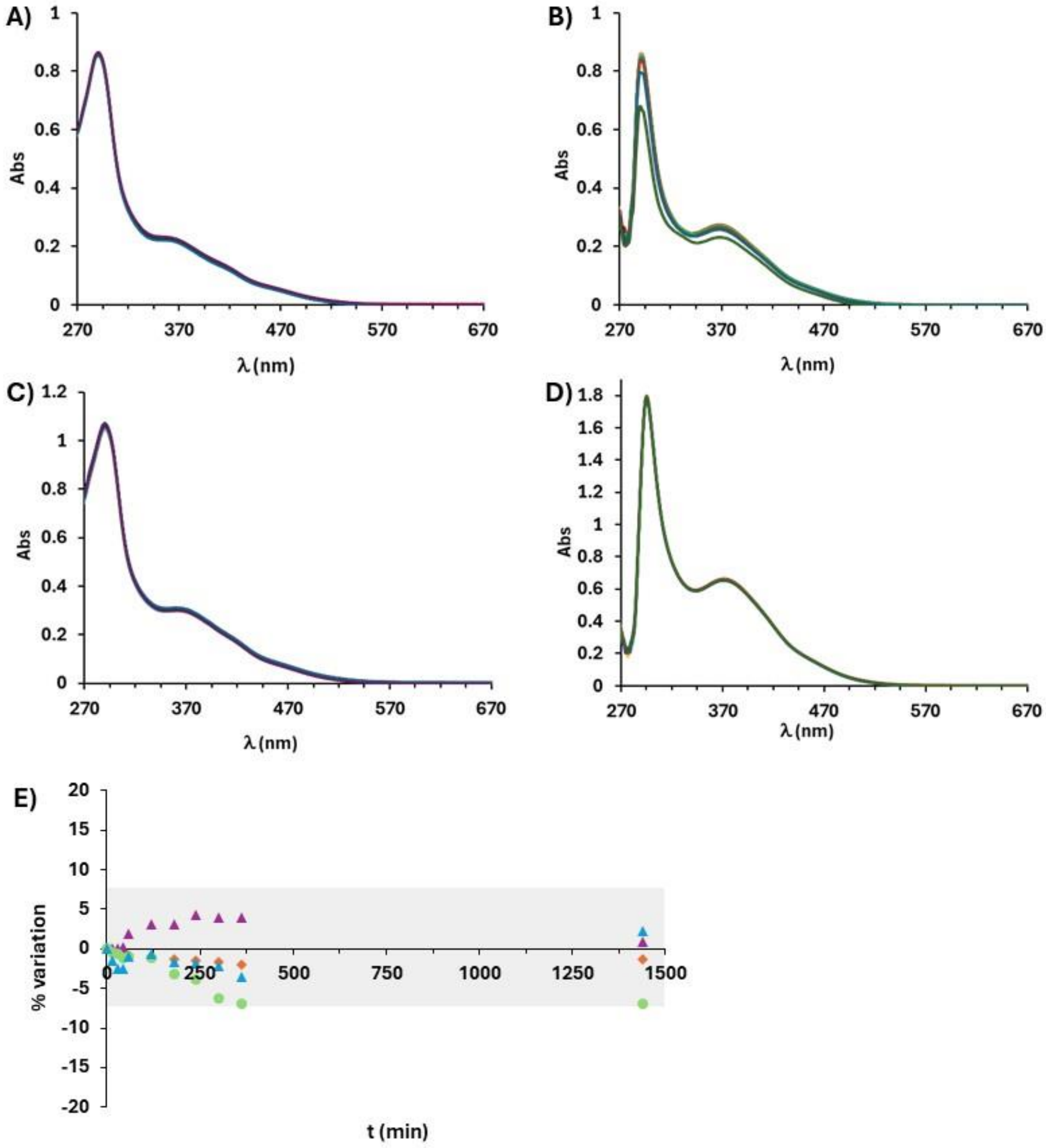

Figure S4. Stability studies followed by UV-Vis spectroscopy over 24 h: (A) compound 1 in DMSO; (B) compound 1 in cellular media (DMSO/DMEM); (C) compound 2 in DMSO; (D) compound 2 in cellular media (DMSO/DMEM). (E) Maximum absorbance variation (%) over time for compound 1 in DMSO (▲ , λ=361 nm); compound 1 in cellular media (●, λ=367 nm); compound 2 in DMSO (♦ , λ=367 nm); compound 2 in cellular media (▲ , λ=368 nm) (see experimental section for details).

NMR SPECTRA

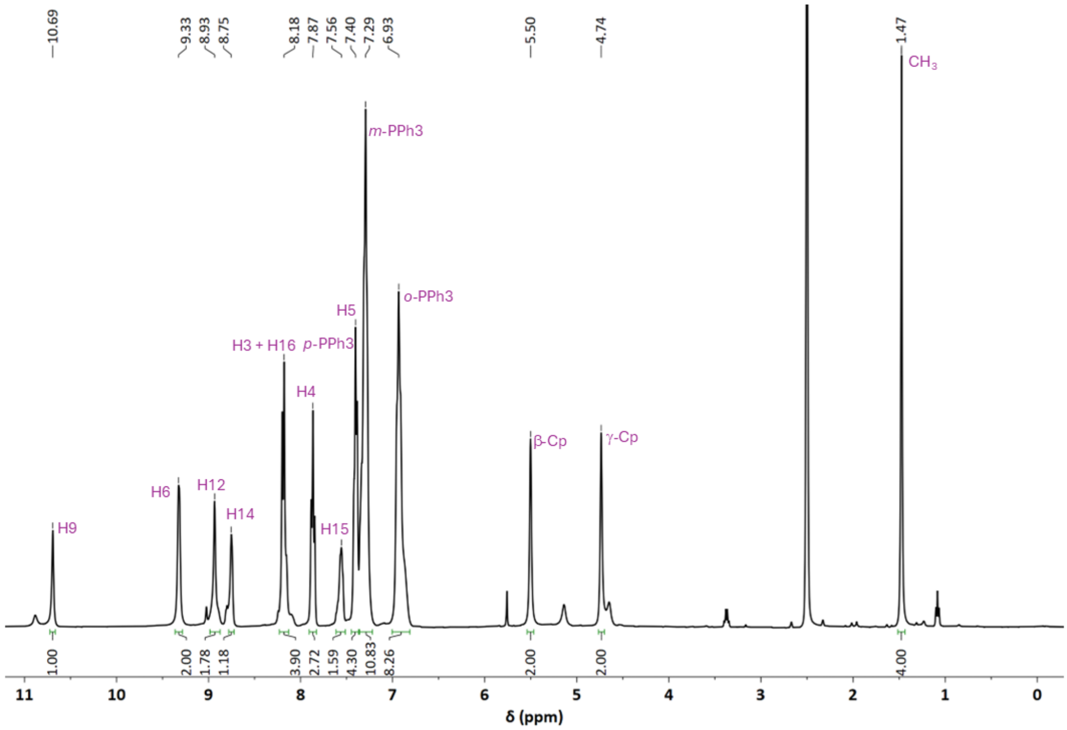

Figure S5. ^{1}H NMR spectrum of [Ru(η^5-$C_5H_4CCH_3$=R1)(PPh_3)(bipy)][CF_3SO_3], R1 = NNHCO(py-3-yl) (**1**) in DMSO-d_6.

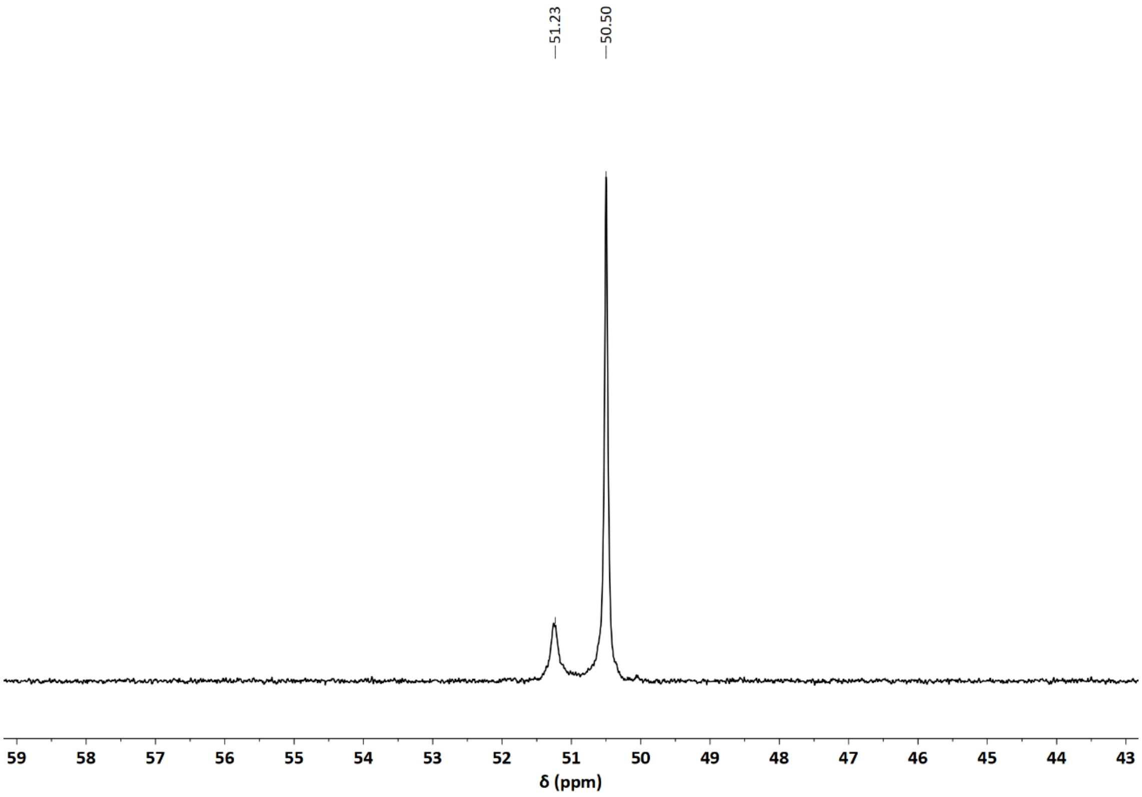

Figure S6. ^{31}P NMR spectrum of [Ru(η^5-$C_5H_4CCH_3$=R1)(PPh_3)(bipy)][CF_3SO_3], R1 = NNHCO(py-3-yl) (**1**) in DMSO-d_6.

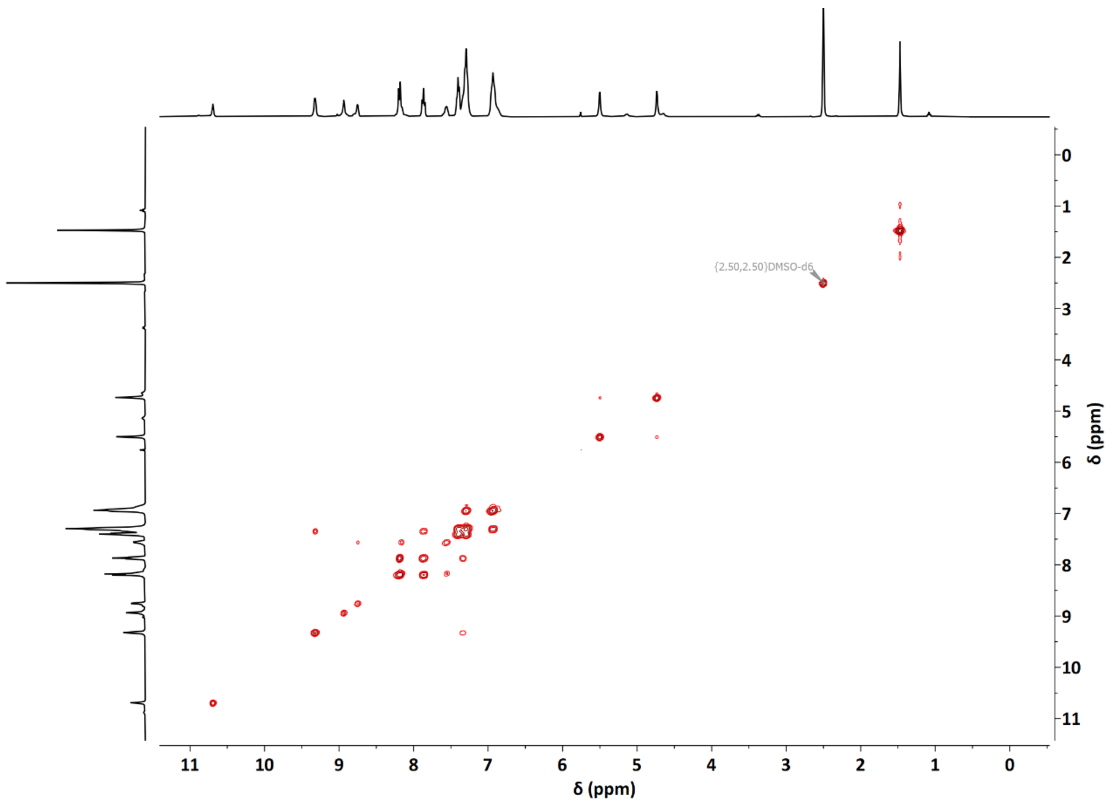

Figure S7. COSY NMR spectrum of [Ru(η^5-$C_5H_4CCH_3$=R1)(PPh_3)(bipy)][CF_3SO_3], R1 = NNHCO(py-3-yl) (**1**) in DMSO-d_6.

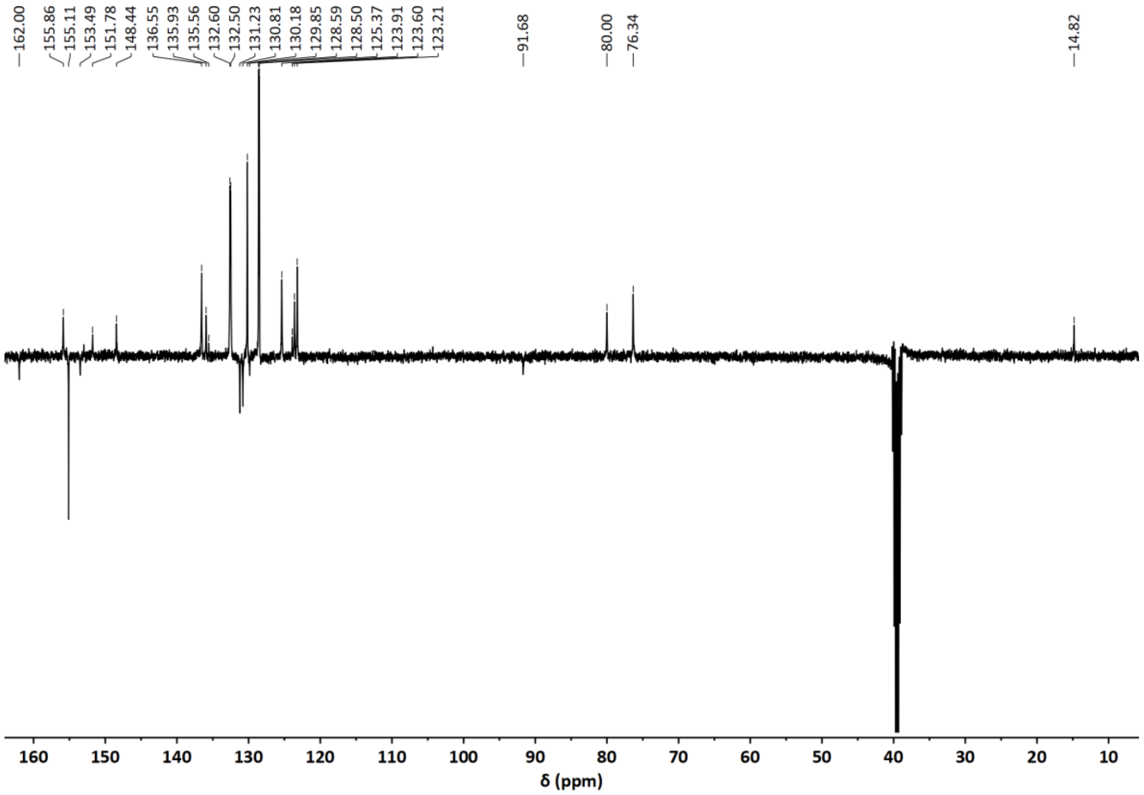

Figure S8. ^{13}C NMR spectrum of [Ru(η^5-$C_5H_4CCH_3$=R1)(PPh_3)(bipy)][CF_3SO_3], R1 = NNHCO(py-3-yl) (**1**) in DMSO-d_6.

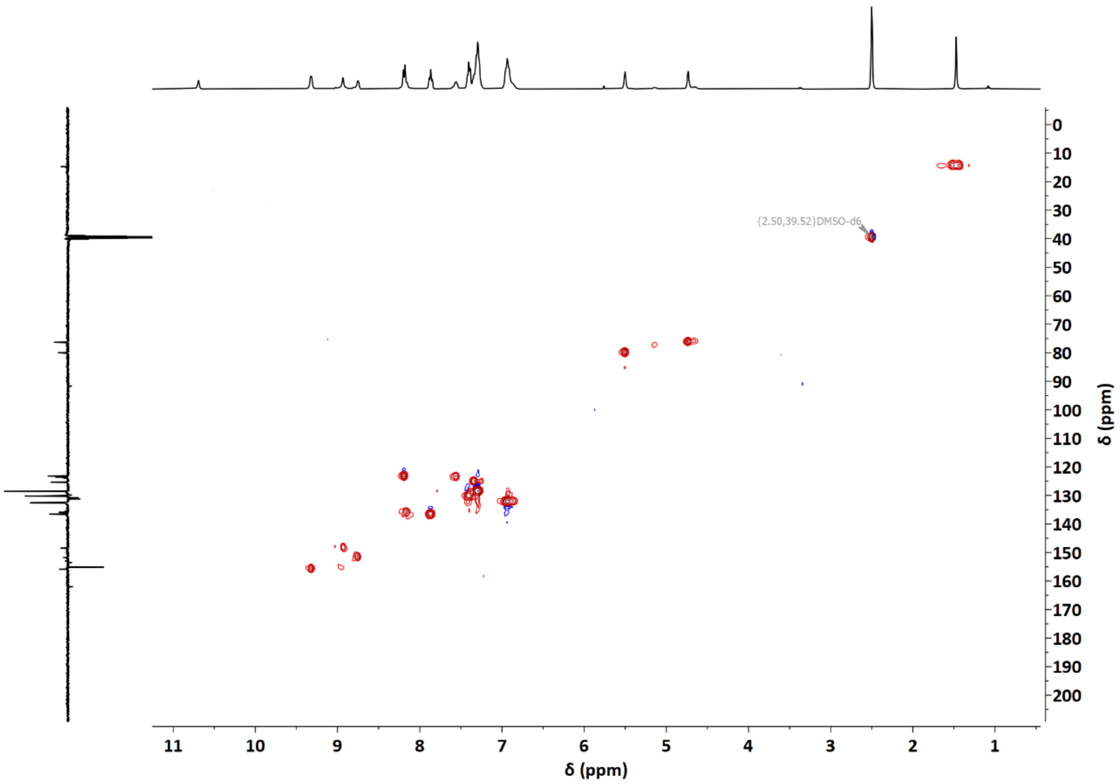

Figure S9. HSQC NMR spectrum of [Ru(η^5-$C_5H_4CCH_3$=R1)(PPh_3)(bipy)][CF_3SO_3], R1 = NNHCO(*py-3-yl*) (**1**) in DMSO-d_6.

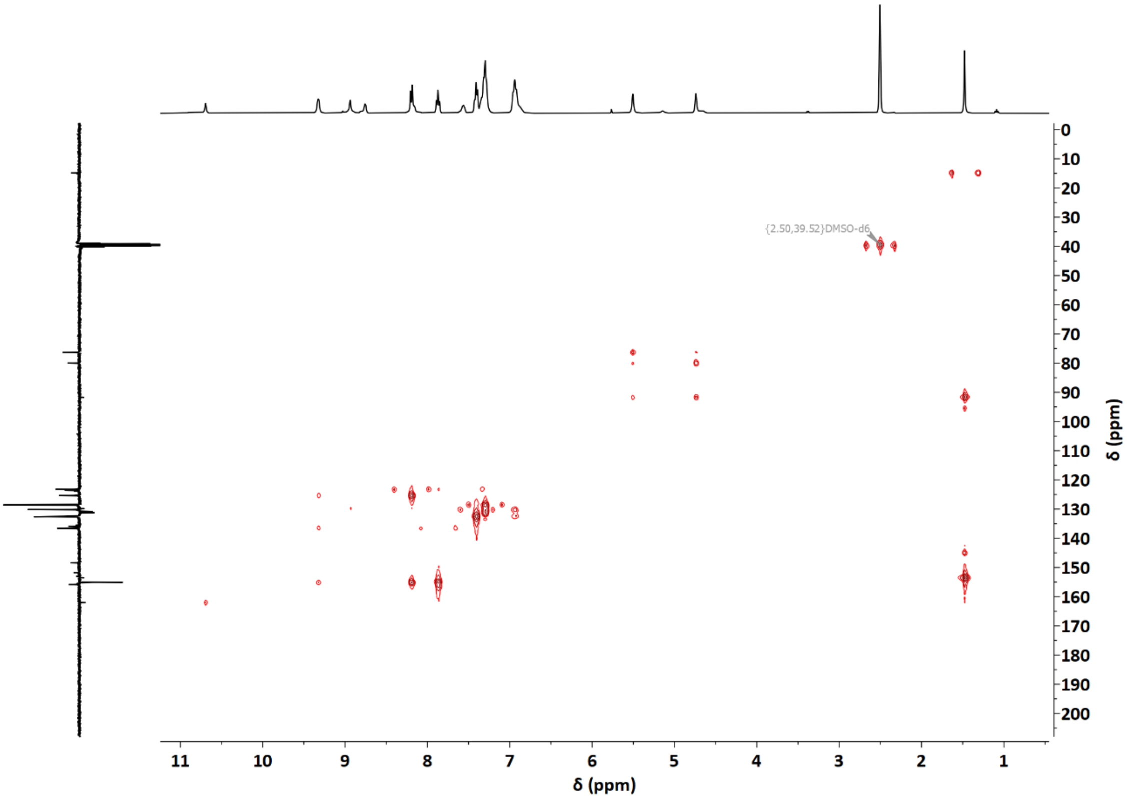

Figure S10. HMBC NMR spectrum of [Ru(η^5-$C_5H_4CCH_3$=R1)(PPh_3)(bipy)][CF_3SO_3], R1 = NNHCO(py-3-yl) (**1**) in DMSO-d_6.

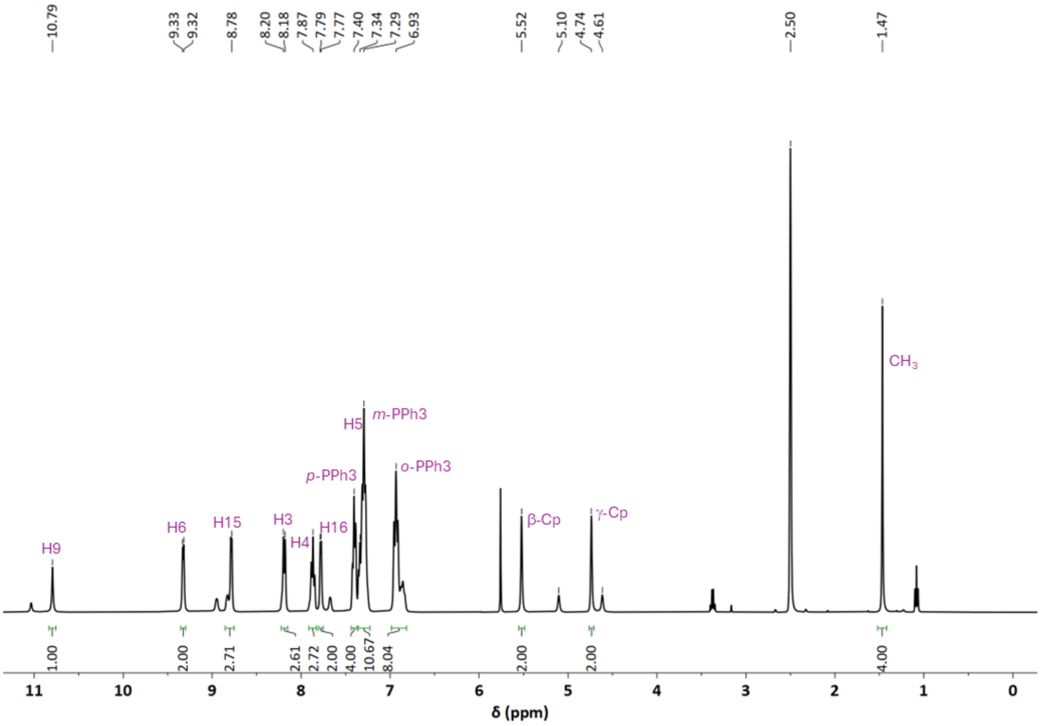

Figure S11. ^{1}H NMR spectrum of [Ru(η^{5}-$C_5H_4CCH_3$=R2)(PPh_3)(bipy)][CF_3SO_3], R2 = NNHCO(py-4-yl) (**2**) in DMSO-d_6.

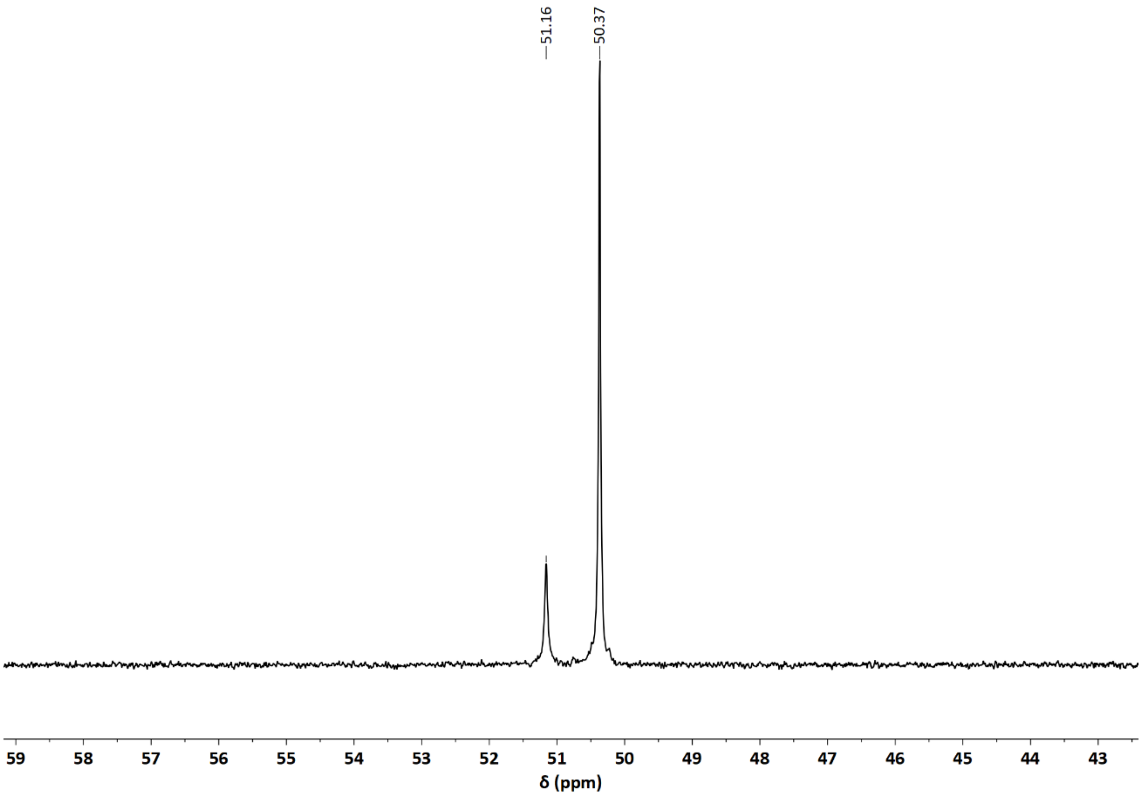

Figure S12. ^{31}P NMR spectrum of [Ru(η^{5}-$C_5H_4CCH_3$=R2)(PPh_3)(bipy)][CF_3SO_3], R2 = NNHCO(py-4-yl) (**2**) in DMSO-d_6.

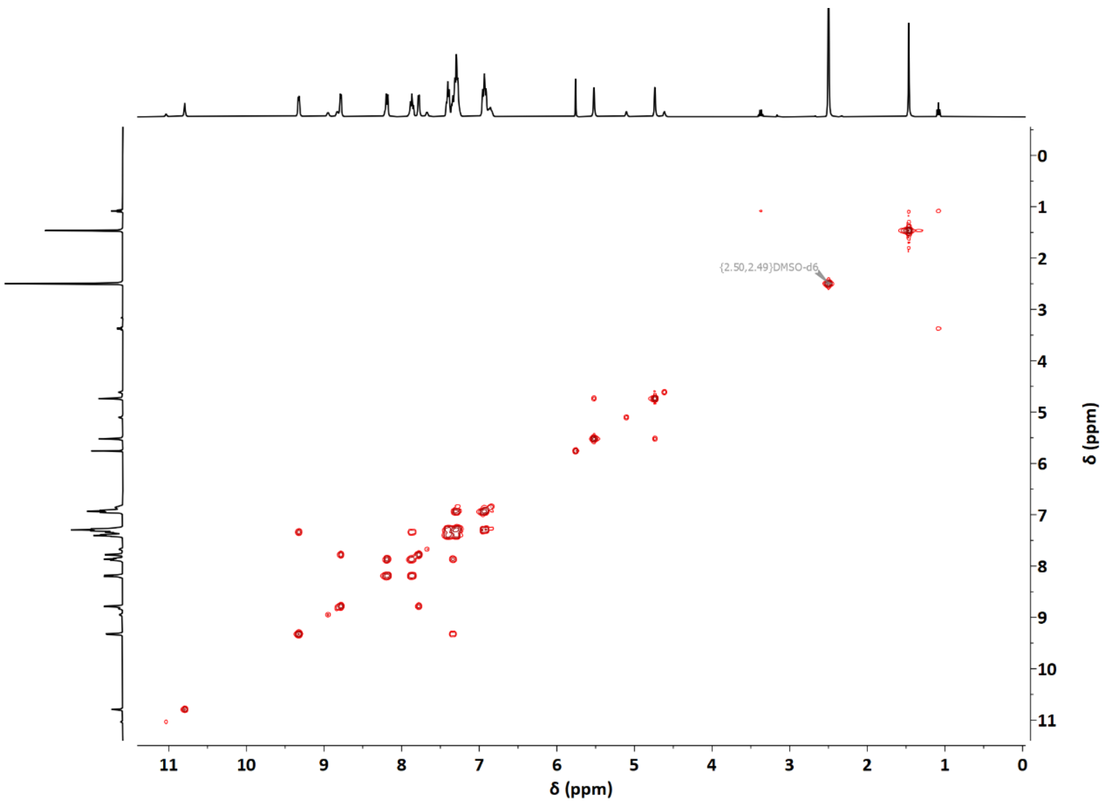

Figure S13. COSY NMR spectrum of [Ru(η^5-$C_5H_4CCH_3$=R2)(PPh_3)(bipy)][CF_3SO_3], R2 = NNHCO(py-4-yl) (**2**) in DMSO-d_6.

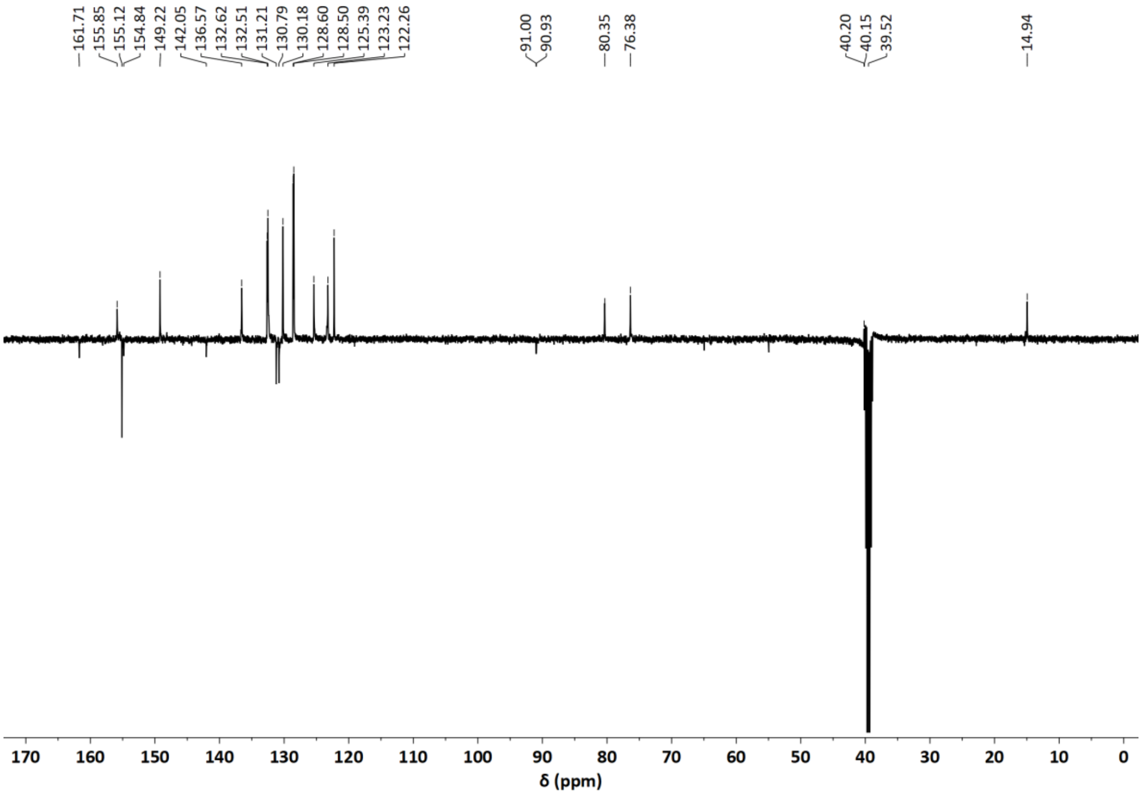

Figure S14. ^{13}C NMR spectrum of [Ru(η^5-$C_5H_4CCH_3$=R2)(PPh_3)(bipy)][CF_3SO_3], R2 = NNHCO(py-4-yl) (**2**) in DMSO-d_6.

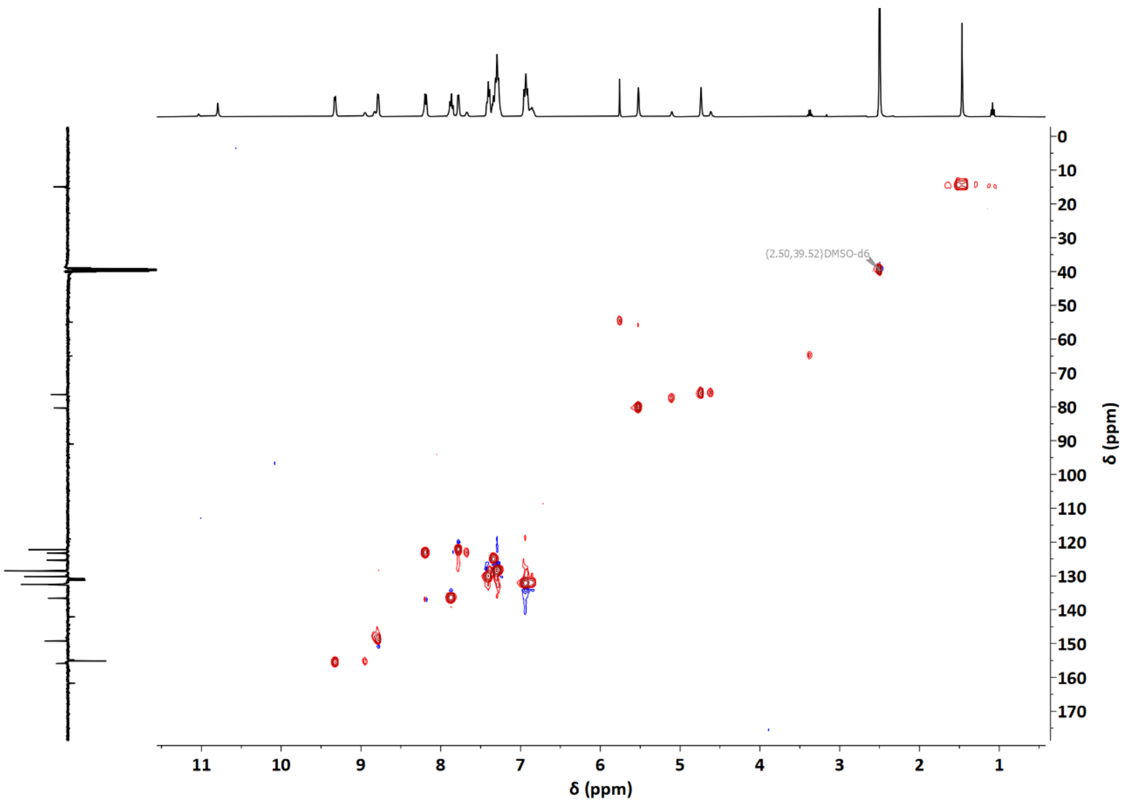

Figure S15. HSQC NMR spectrum of [Ru(η^5-$C_5H_4CCH_3$=R2)(PPh_3)(bipy)][CF_3SO_3], R2 = NNHCO(py-4-yl) (**2**) in DMSO-d_6.

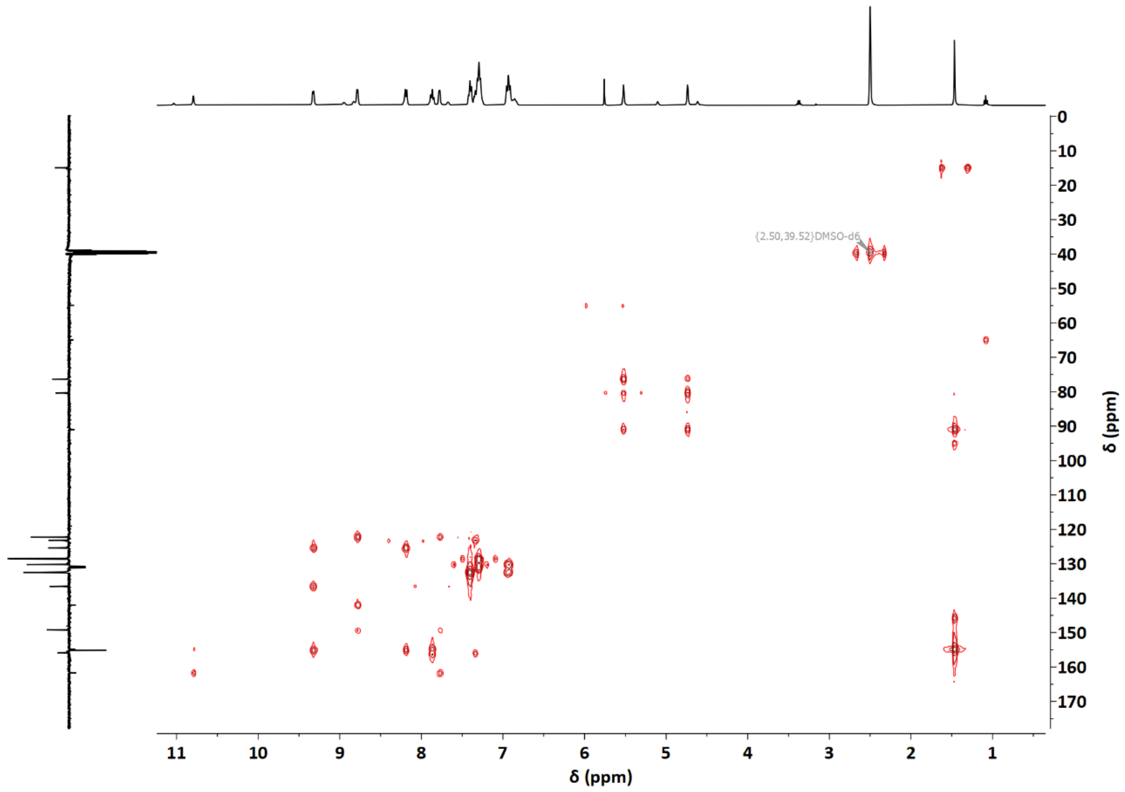

Figure S16. HMBC NMR spectrum of [Ru(η^5-$C_5H_4CCH_3$=R2)(PPh_3)(bipy)][CF_3SO_3], R2 = NNHCO(py-4-yl) (**2**) in DMSO-d_6.

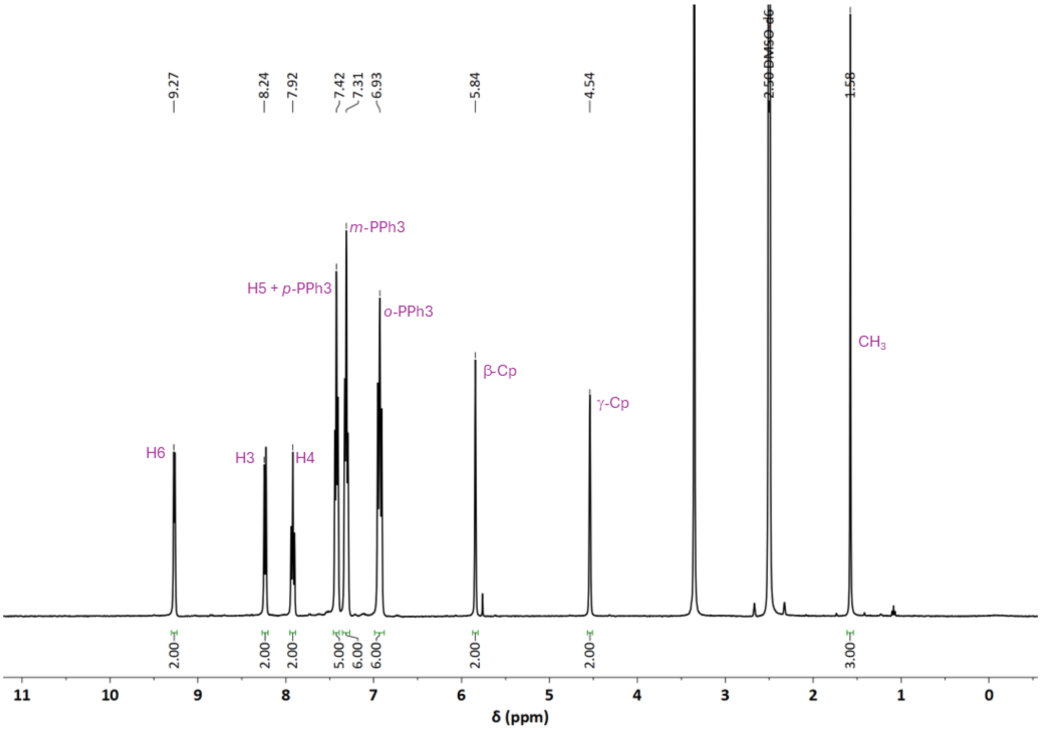

Figure S17. ^{1}H NMR spectrum of $[Ru(\eta^5-C_5H_4CH_3)(PPh_3)(bipy)][CF_3SO_3]$ in DMSO-d_6.

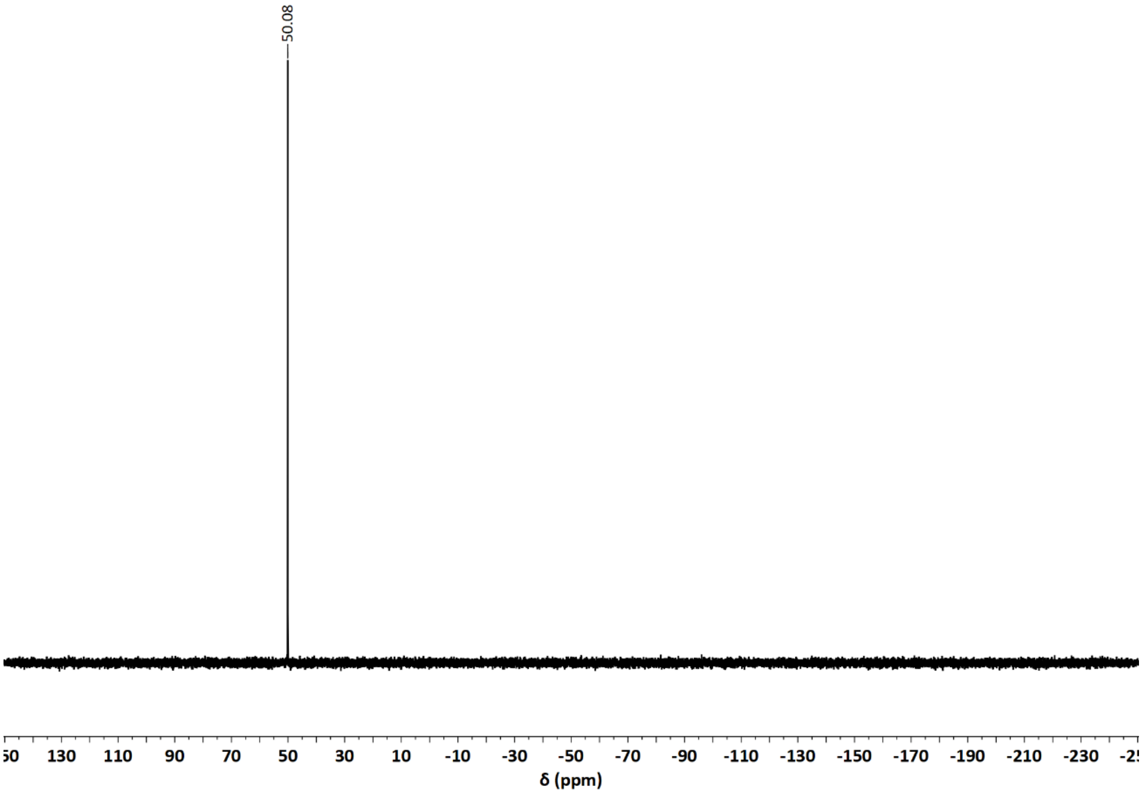

Figure S18. ^{31}P NMR spectrum of $[Ru(\eta^5-C_5H_4CH_3)(PPh_3)(bipy)][CF_3SO_3]$ in DMSO-d_6.

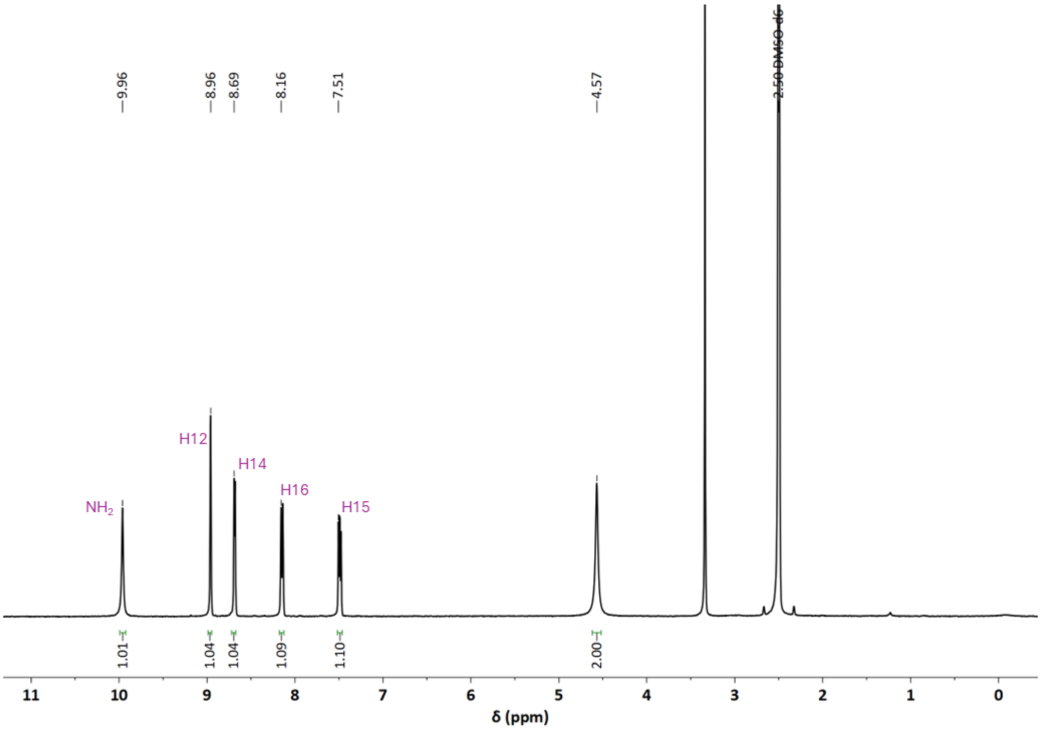

Figure S19. ^{1}H NMR spectrum of NAH in DMSO-d6.

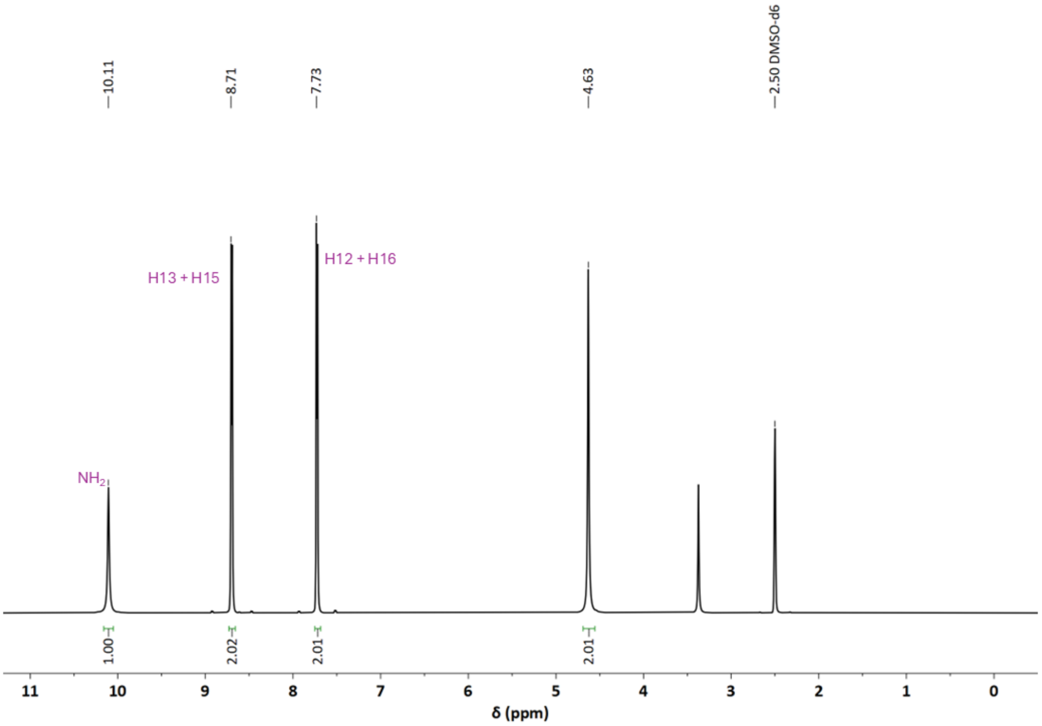

Figure S20. ^{1}H NMR spectrum of INH in DMSO-d6.

FT-IR SPECTRA

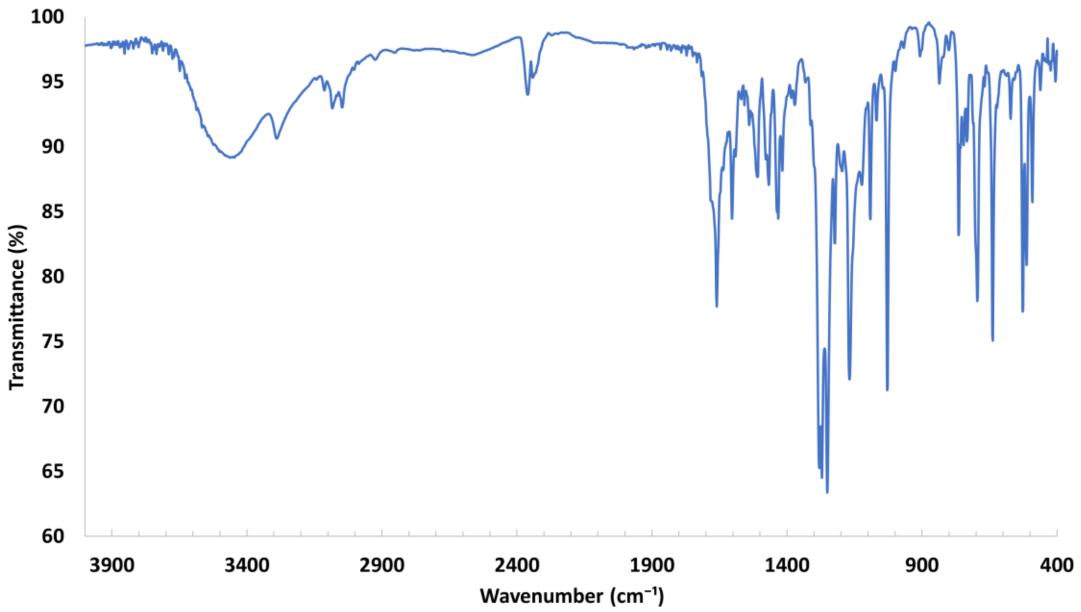

Figure S21. FTIR spectrum of [Ru(η^5-$C_5H_4CCH_3$=R1)(PPh_3)(bipy)][CF_3SO_3], R1 = NNHCO(py-3-yl) (**1**) in KBr.

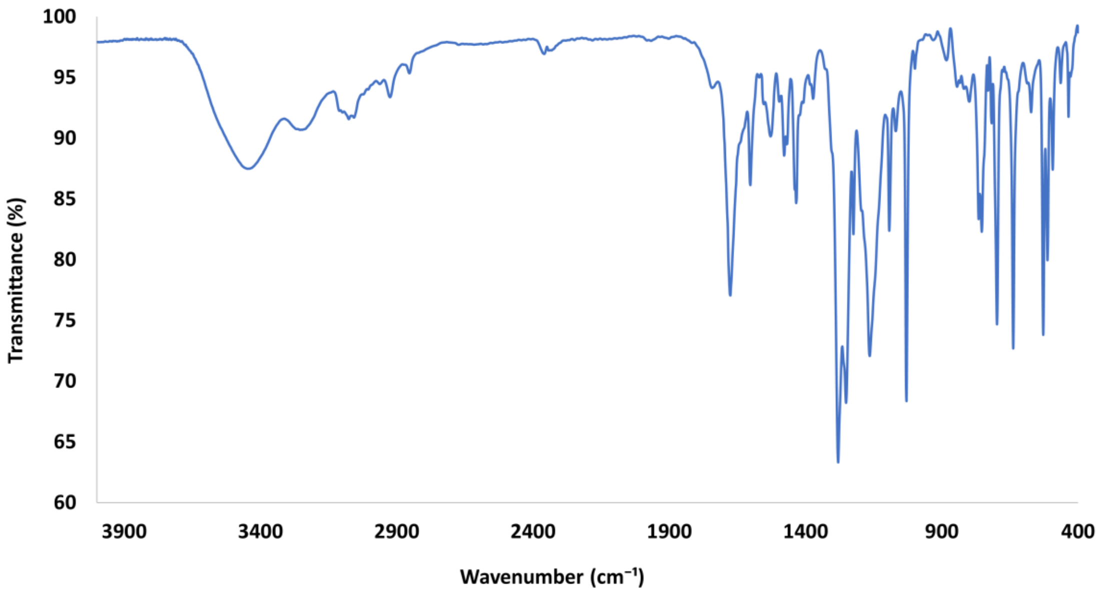

Figure S22. FTIR spectrum of [Ru(η^5-$C_5H_4CCH_3$=R2)(PPh_3)(bipy)][CF_3SO_3], R2 = NNHCO(py-4-yl) (**2**) in KBr.

HRMS(ESI-MS) SPECTRA

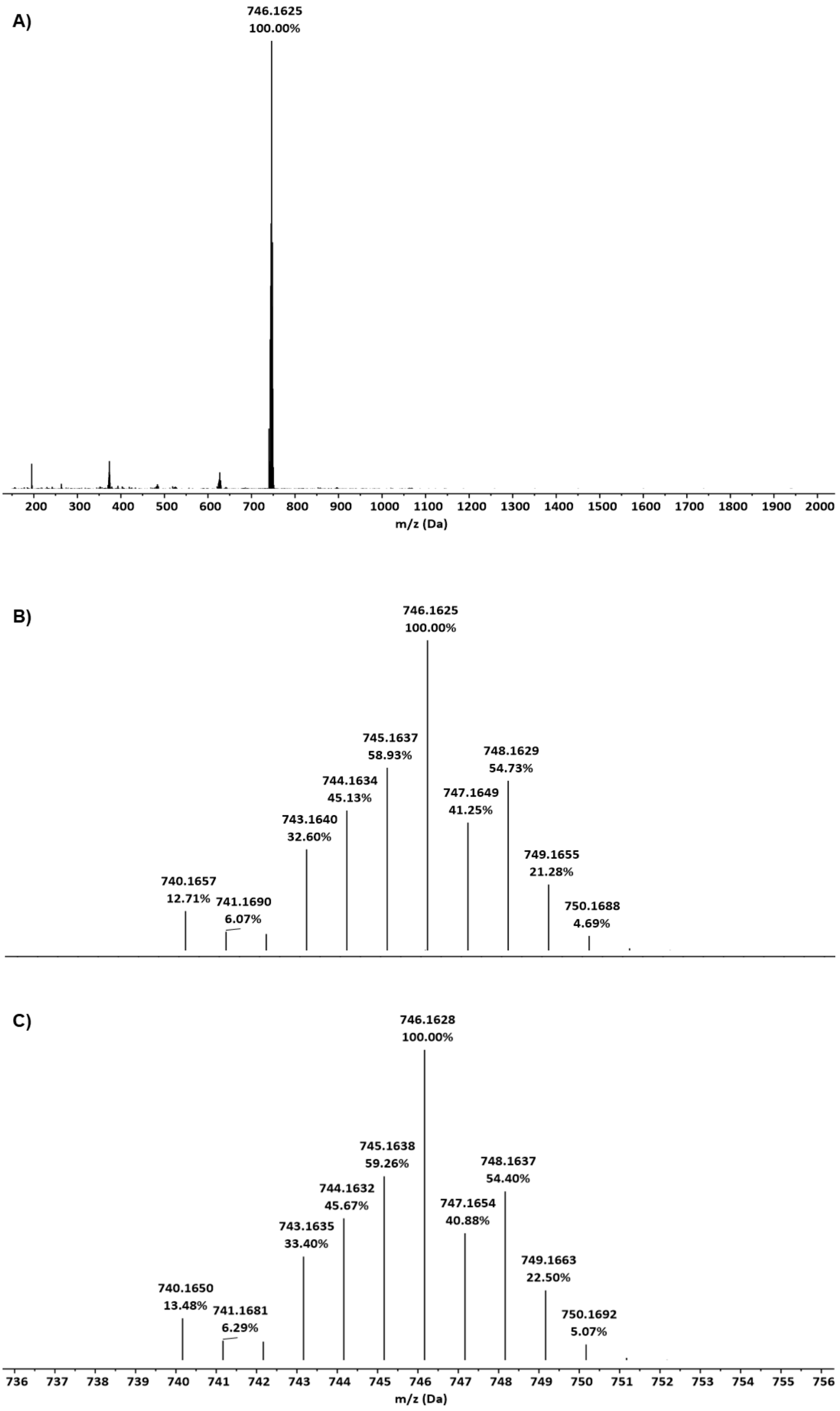

Figure S23. (A) High-resolution ESI-MS spectrum of complex 1; Calculated (B) and found (C) isotopic pattern

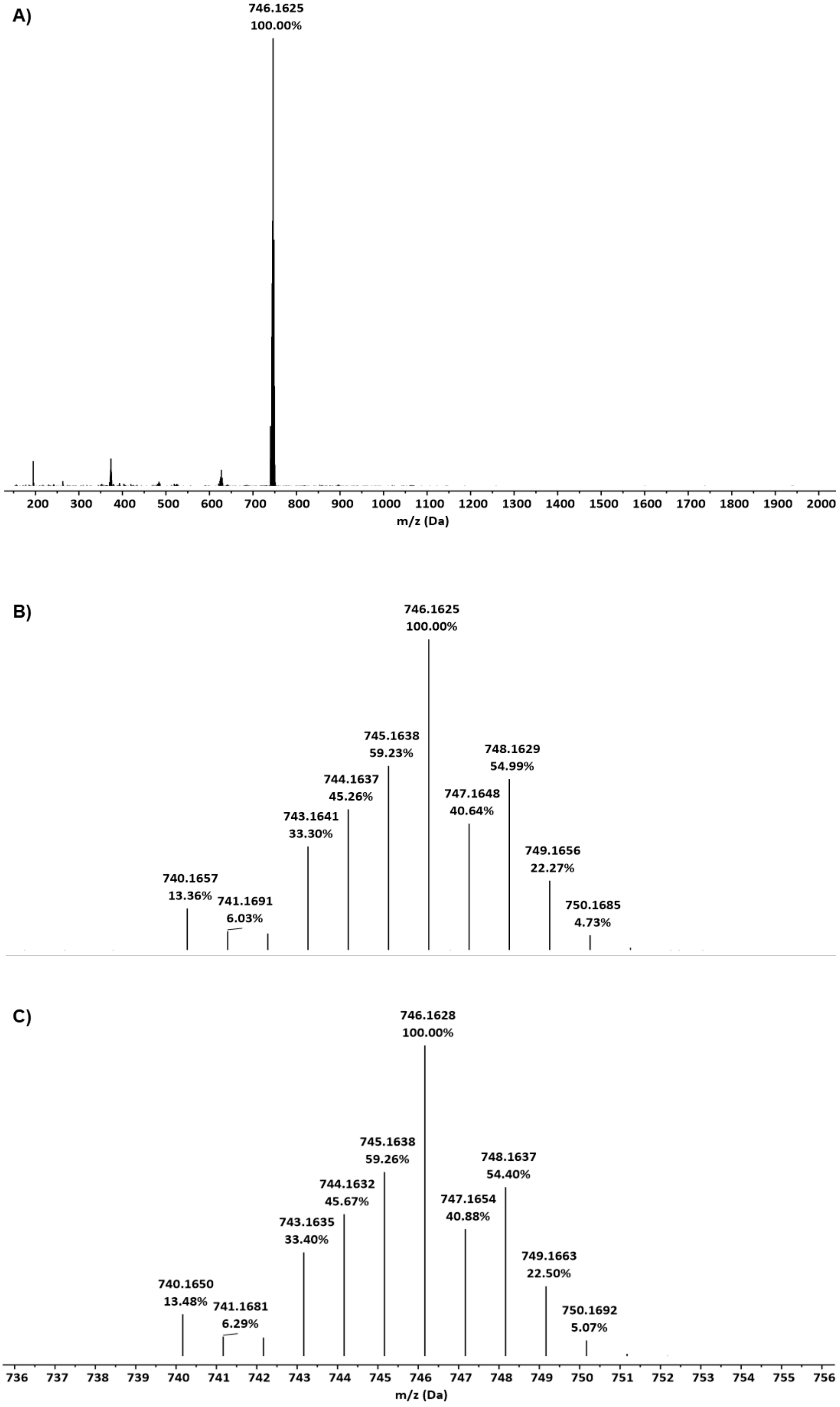

Figure S24. (A) High-resolution ESI-MS spectrum of complex 2; Calculated (B) and found (C) isotopic pattern
